# Personalized Prediction of Proliferation Rates and Metabolic Liabilities in Cancer Biopsies

**DOI:** 10.3389/fphys.2016.00644

**Published:** 2016-12-27

**Authors:** Christian Diener, Osbaldo Resendis-Antonio

**Affiliations:** ^1^Human Systems Biology Laboratory, Instituto Nacional de Medicina GenómicaMéxico City, Mexico; ^2^Coordinación de la Investigación Científica, Red de Apoyo a la Investigación, UNAMMéxico City, Mexico

**Keywords:** systems biology, personalized medicine, proliferation, flux balance analysis, TCGA, NCI60

## Abstract

Cancer is a heterogeneous disease and its genetic and metabolic mechanism may manifest differently in each patient. This creates a demand for studies that can characterize phenotypic traits of cancer on a per-sample basis. Combining two large data sets, the NCI60 cancer cell line panel, and The Cancer Genome Atlas, we used a linear interaction model to predict proliferation rates for more than 12,000 cancer samples across 33 different cancers from The Cancer Genome Atlas. The predicted proliferation rates are associated with patient survival and cancer stage and show a strong heterogeneity in proliferative capacity within and across different cancer panels. We also show how the obtained proliferation rates can be incorporated into genome-scale metabolic reconstructions to obtain the metabolic fluxes for more than 3000 cancer samples that identified specific metabolic liabilities for nine cancer panels. Here we found that affected pathways coincided with the literature, with pentose phosphate pathway, retinol, and branched-chain amino acid metabolism being the most panel-specific alterations and fatty acid metabolism and ROS detoxification showing homogeneous metabolic activities across all cancer panels. The presented strategy has potential applications in personalized medicine since it can leverage gene expression signatures for cell line based prediction of additional metabolic properties which might help in constraining personalized metabolic models and improve the identification of metabolic alterations in cancer for individual patients.

## Introduction

Cancer is a heterogeneous disease that manifests in a wide variety of geno- and phenotypes. There is no one treatment that works for any cancer types and even cancers of the same phenotype may show large genomic or metabolic differences (Hu et al., 2013; Andor et al., 2015; Hensley et al., 2016). Due to this, there has been an ongoing effort to characterize the particular signatures of cancer in the genome and transcriptome (Mazor et al., 2016; Tirosh et al., 2016) and elucidate its tissue specific consequences for cancer patients. Two of the largest projects describing genomic and expression features of several cancers are the NCI60 and TCGA projects (Scherf et al., 2000; Shoemaker, 2006; Koboldt et al., 2012; Zheng et al., 2016). Currently, NCI60 comprises 60 cancer cell lines and their full genetic, transcriptomic and proteomic characterization. The Cancer Genome Atlas project has similar goals but for cancer samples coming from several thousand patients. Detailed studies of those data sets have revealed the variation inherited even within a single cancer panel and provide great potential for uncovering of the genomic differences that drive the strong variability in cancer phenotypes (Hoadley et al., 2014).

The NCI60 and TCGA databases concentrate on genomic characterizations of distinct cancers which creates the challenge to connect those data to metabolism, which itself is closely connected to the cancer phenotype by providing the macromolecules required for proliferation (Boroughs and DeBerardinis, 2015). Here, the cell lines contained in NCI60 have been characterized in more detail by providing the proliferation rates for the majority of the 60 cancer panels (in the form of doubling times). Due to the inherent complications in measuring those quantities in patients, TCGA includes clinical indicators but lacks biological characterizations of the cancer samples outside of genomic data. In particular, TCGA lacks quantification of cancer proliferation.

In general, inference of metabolic properties from genome and gene expression data is a difficult task due to the many post-transcriptional and post-translational regulatory mechanisms involved in central carbon metabolism that are usually not fully captured by sheer mRNA or protein abundance. Consequently, there have been many attempts to infer the metabolic state by computational methods. Here, flux balance analysis (FBA) is the most prominent one and has proven to be helpful in the analysis of cancer metabolism in cell lines and tissue-specific metabolic models (Orth et al., 2010; Resendis-Antonio et al., 2010; Agren et al., 2014; Yizhak et al., 2015). There are several algorithms performing this task but they all aim to reconcile gene expression or proteome data with the presence of distinct biochemical reactions in the model in some way or another (Becker and Palsson, 2008; Agren et al., 2012; Wang et al., 2012). The major limit to those models are the lack of metabolic data and the weak association between enzyme expression and metabolic fluxes. Due to this, many of the reconstruction methods use discretized enzyme expression values in order to exclude biochemical reactions with a lacking enzyme (Wang et al., 2012; Pornputtapong et al., 2015; Schultz and Qutub, 2016). This strategy has shown to be a promising approach in constraining the feasible metabolic space in cells or tissues and predicting the metabolic capacities of several cancers (Agren et al., 2014). One of the challenges in using FBA-based methods is finding sufficient constraints to identify the unique set of metabolic fluxes for a biological sample. Here, parsimonious FBA, where one only uses the most economic flux distribution for a metabolic objective, has shown to reproduce experimentally measured fluxes and may in some cases even outperform methods based on gene expression data (Lewis et al., 2010; Machado and Herrgård, 2014). Furthermore, it has also been shown that knowledge of the associated proliferation rate will yield to an improvement of those predictions making it desirable to complement expression data with at least a limited set of fluxome data such as growth rates or measurements of key fluxes (Yizhak et al., 2014). Growth rates for simpler eukaryotes can be predicted from gene expression signatures (Airoldi et al., 2009), thus raising the question whether one can identify growth or proliferation rates for clinical samples from gene expression data.

The combination of genome-scale metabolic modeling, personalized reconstruction, and inference of additional metabolic constraints forms the core of a strategy that shows high promises in personalized medicine. Here, accurate prediction of metabolic fluxes may help to identify distinct metabolic alterations and the causality underlying diseases in individual patients by identifying a patient-specific set of altered metabolic processes (Bordbar et al., 2015; Resendis-Antonio et al., 2015).

In this work we present a strategy capable of predicting proliferation rates for more than 12,000 cancer samples in the Cancer Genome Atlas by training a machine learning model for proliferation on the NCI60 data set. We show that the predicted proliferation rates correspond well with clinical data and employ them to estimate the fluxes driving cancer proliferation for more than 3500 samples from nine different cancer subtypes. Overall, our study provides a computational strategy that is able to predict the proliferation rate of cancer biopsies from cell line gene expression data alone and this allows detailed surveys of the potential metabolic activity underlying each case. As a result, our methodology can contribute to the identification of the common and specific metabolic alterations associated with cancers across different tissues, which is of importance during the development of personalized treatments for cancer.

## Data and methods

### Data availability and software

All source code and additional data needed to run the analysis is hosted on GitHub in a dedicated paper repository at https://github.com/cdiener/proliferation and is archived by Zenodo (http://doi.org/10.5281/zenodo.166813). We also provide intermediate data sets for the NCI60 (http://doi.org/10.5281/zenodo.61980) and TCGA data (http://doi.org/10.5281/zenodo.61982). The repository includes Rmarkdown documents (http://rmarkdown.rstudio.com/) detailing the exact steps to produce the reported results and this information is also contained in the Supplementary Protocol S1 in PDF format. Respective software versions are reported in Protocol S1 under “Software versions.” We also provide a docker image in order to reproduce our entire analysis interactively on a local machine or in the cloud at https://hub.docker.com/r/cdiener/proliferation.

### NCI60 and TCGA data sets

HuEx ST 1.0 gene expression data for the NCI60 cancer cell lines was obtained from the GEO database from experiment GSE29682 (Reinhold et al., 2010; Barrett et al., 2013). The data was read using the oligo package from Bioconductor and normalized by RMA (Carvalho and Irizarry, 2010). This was followed by a summary step where we calculated the expression for each gene in each sample as the mean log expression across all probesets that were mapped to this gene. Here, the probeset-gene mapping was obtained from biomart (http://www.biomart.org) and is also provided in the paper repository (Smedley et al., 2015). Finally, replicates for a given cell line were summarized again by obtaining the mean log expression values across all replicates for a given cell line and gene.

TCGA data was obtained and parsed from the NCI Genomic Data Commons (GDC) repository (see https://gdc-portal.nci.nih.gov/). HuEx 1.0 ST data was obtained from the GDC legacy archive (https://gdc-portal.nci.nih.gov/legacy-archive). Download and parsing was performed in an automated manner using the tcgar package for the R programming language (https://github.com/cdiener/tcgar) which we created for that purpose. A complete list of downloaded files can be found in the “GDC” subfolder of the data repository (https://github.com/cdiener/proliferation). All analysis was based on Level 3 data (already preprocessed data) since this subset available to the general public.

### Generalized linear models

Generalized linear models were fitted using the glmnet package for R (Friedman et al., 2010). Regularization was performed using the L1 norm where the regularization strength λ was chosen as the one yielding the smallest mean squared error during cross-validation. In order to improve regularization we also discarded very small coefficients in the final step of feature selection. Thus, for the final model we included only coefficients larger than the 25% quartile of the non-zero absolute coefficients (see Protocol S1). The resulting fits were analyzed using a set of 5 metrics, namely mean squared error (mse), root mean squared error (rmse), mean absolute error (mae), mean relative error (mre) and *R*^2^. Those metrics were calculated for the training set as well as for leave-one-out cross validation. Here, predictive power was evaluated by the leave-one-out cross validation alone.

### Flux analysis

Flux analysis was performed using the Python programming language (https://python.org) and the COBRApy package (Ebrahim et al., 2013). Metabolic models were obtained from the Human Metabolic Atlas (https://metabolicatlas.org) using the available cancer models which contain a proliferation objective function (Gatto et al., 2014). Given the predicted proliferation rates r_*p*_, fluxes for the models were obtained by parsimonious flux balance analysis (pFBA) by first splitting each reversible reaction into its forward and backward reaction and then solving the resulting linear programming problem for each sample (Lewis et al., 2010):

(1) $$\begin{array}{c} Minimize\sum_{i}v_{i}\\ Sv=0\\ v_{i}\geq 0\\ v_{p}=r_{p} \end{array}$$

Here, S denotes the stoichiometric matrix of the respective irreversible metabolic model, *v*_*i*_ denotes the flux with index *i* and *v*_*p*_ denotes the the flux of the proliferation objective. Note that, given the proliferation rate *r*_*p*_ this does not require constraints for the fluxes other than positivity. Given the large number of optimization problems we employed a strategy similar to FastFVA during optimization where each optimization was performed once *de novo* for each model and subsequent optimizations on the same model recycled the previous solution basis which allows for fast computation of the fluxes (Gudmundsson and Thiele, 2010). Optimization was only performed for samples with a positive proliferation rate and we only used fluxes in further analysis which were non-zero for at least one sample, yielding a total of 1026 used fluxes.

Specificity for a given cancer subtype was scored for each flux as the relative difference of the mean flux within the cancer panel vs. all other cancer panels.

(2) $$s_{p}^{i}={\text{log}}_{\text{2}}\mu_{p}^{i}-{\text{log}}_{\text{2}}\mu_{o}^{i}$$

Here $\mu_{p}^{i}$ denotes the mean of flux *v*_*i*_ across all samples in cancer panel p and $\mu_{o}^{i}$ the mean of flux *v*_*i*_ in all other samples. Thus, the resulting specificity score $s_{p}^{i}$ described the log-fold change of the target flux between the target cancer panel and the rest of all the samples.

Pathway enrichment was obtained by using an enrichment score similar to GSEA (Mootha et al., 2003; Subramanian et al., 2005). First, specificity scores $s_{p}^{i}$ were sorted from highest to lowest absolute value across all panels and fluxes, yielding the ranked list *R* containing n elements. Then we calculated a raw enrichment score for a metabolic pathway mp mapping to *n*_*h*_ elements in *R* as

(3) $$\begin{array}{l} \;ES=max_{i}/min_{i}\;P_{h}\left(i\right)-P_{m}\left(i\right)\\ \;where\;P_{h}\left(i\right)=\sum_{v_{i}\;\in \;mp,\;j\;\leq \;i}R_{j}/n_{r},n_{r}=\sum_{j\;\in \;mp}R_{j}\\ and\;P_{m}\left(i,mp\right)=\sum_{v_{i}\;not\;\in \;mp,\;j\;\leq \;i}1/\left(n-n_{h}\right) \end{array}$$

ES will be large when the respective pathway is enriched in the beginning of *R* (specific fluxes are enriched in the pathway), and will be negative when the the pathway occurs in the tail of *R* (specific fluxes are depleted in the pathway). The score was then normalized by randomly permuting the pathway labels 100 times for each pathway, obtaining the respective mean permuted enrichment score ES_perm_, and calculating the normalized enrichment score as NES = ES/ES_perm_. Empirical p-values for the normalized enrichment scores were obtained from the 100 random permutations separately for the positive and negative tails. Thus, the normalized enrichment score NES denotes the fold change between the real pathway mapping and a randomly generated one. If NES is larger than one this denotes an enrichment of the given pathway in the specific fluxes, whereas a NES smaller than one denotes absence of the given pathway in the specific fluxes. Hence, NES > 1 identifies metabolic pathways that are active in cancer cell panel-specific manner whereas NES <1 identifies metabolic pathways that are underrepresented in the panel-specific fluxes and thus form a set of core pathways whose activity does not vary across the cancer panels.

## Results

### Identification of stable gene signatures across technologies and cell types

One of the major challenges when studying two large data sets such as NCI60 and TCGA together is the conservation of gene expression across different technologies and cell types. In the NCI60 data set gene expression was measured by microarrays with the HuEx 1.0 ST arrays being the most recent technology used. TCGA however mostly used RNA-seq for the quantification of gene expression and provides microarray data for only a small subset of cancer panels. For instance, TCGA includes HuEx 1.0 ST data for 1211 samples across 3 cancer panels but RNA-seq data for 11,093 samples across all 33 cancer panels. In order to include the majority of cancer panels in TCGA into the analysis, we thus tried to identify a subset of genes that showed similar global expression across NCI60 and TCGA. We first obtained the mean log expression values for all genes contained in the NCI60 HuEx 1.0 ST data as well as in the TCGA RNA-seq and HuEx 1.0 ST data. For the NCI60 data set this mean log expression was calculated across all cell lines for which proliferation rates were available (57 of 60), whereas the mean log expression for the TCGA data set was obtained by averaging over all samples.

**Figure 1:**
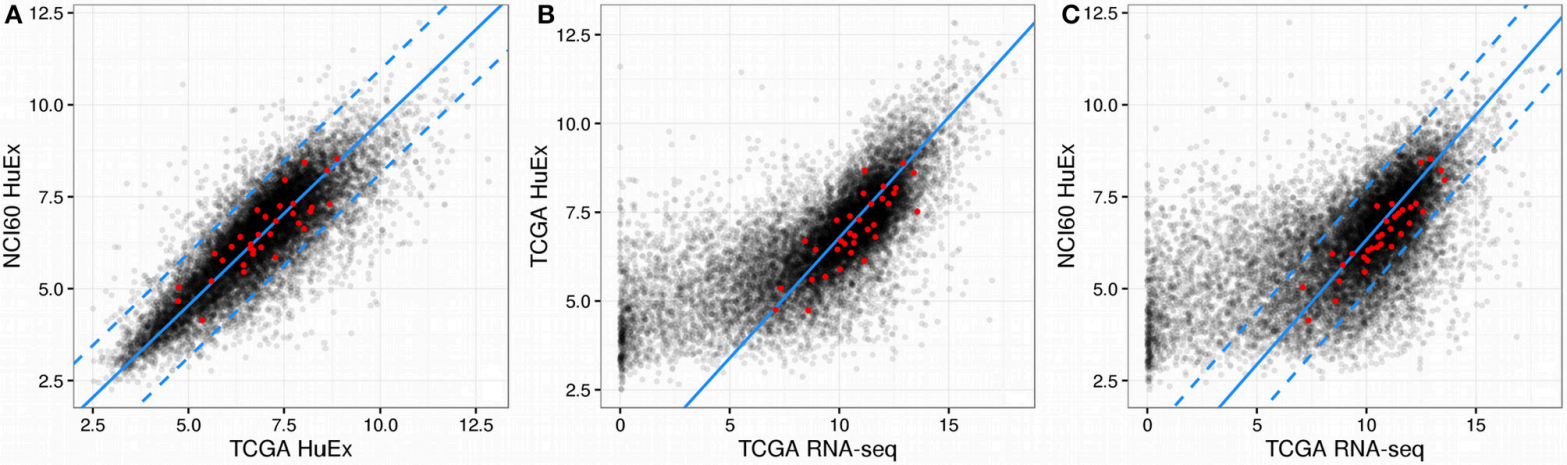
**Gene expression across NCI60 and TCGA**. In all figures the red dots denote the gene that were used in the final predictor for proliferation rates and dashed lines enclose the area used for filtering viable gene candidates. **(A)** HuEx expression data cross NCI60 and TCGA. The blue solid line denotes a 1:1 relationship offset by the parameter beta. **(B)** Gene expression between microarray and RNA-Seq data within TCGA. The solid blue line denotes the slope given by alpha and passes through the origin. **(C)** Gene expression between microarray and RNA-Seq data across NCI60 and TCGA. The solid blue line is given by the slope alpha and intercept beta which were obtained individually from the data shown in **(A,B)**.

Within the NCI60 and TCGA sample subsets that were measured by the HuEx microarrays sets expression values were similar (correlation 0.82, *p* < 2.2e-16, compare Figure 1A), indicating that the used cell lines are an adequate model system for human cancer cells. Comparing the microarray log expression values from NCI60 to RNA-seq log expression values from TCGA we found a more complex relation. Here, genes that showed a high expression in the RNA-seq data showed a linear relationship with the NCI60 microarray log expression values (compare Figure 1C). However, most of the genes with low expression in the TCGA RNA-seq data showed almost random expression values in the NCI60 HuEx data and a similar behavior could be observed when comparing the TCGA microarray data with the TCGA RNA-seq data (see Figure 1B). There are several possible explanations for this discrepancy, such as a the low dynamic range of microarrays, cell line-specific expression of some genes, or technical errors. Thus, we aimed at selecting only those genes that showed a globally correlated expression between the NCI60 microarray data and the TCGA RNA-seq data. Genes, whose expression was conserved across both platforms were identified by a linear model relating mean log gene expression values from the NCI60 HuEx experiments ($e_{N}^{i}$) and and the TCGA RNA-Seq experiments ($e_{T}^{i}$) as

(4) $$e_{T}^{i}=\alpha e_{N}^{i}+\beta$$

Here, α denotes a platform-specific factor that describes the mapping from microarray to RNA-seq expression values for the same samples, whereas β denotes a sample parameter which adjusts for different sample quantities between the NCI60 and TCGA data set. One could fit those parameters directly using the NCI60 Huex and TCGA RNA-Seq data, however, we chose to use a more robust approach in which each of the two parameters was obtained individually from other data set combinations. Here, α was obtained by fitting the HuEx and RNA-Seq data contained in TCGA to a zero-intercept linear model (same samples implies β = 0), whereas β could be obtained by calculating the difference between the mean log expression values of the HuEx data from NCI60 and TCGA (same platform implies α = 1). The full model was then validated using the NCI60 Huex and TCGA RNA-Seq and showed good agreement with the data as is shown in Figure 1. As a consequence the fitted model could be used to correct the NCI60 log expression values to its respective TCGA RNA-seq log expression value.

Following the model fit, genes with conserved expression across both data set could be obtained by enforcing the linear relationship described before. In detail, genes were considered acceptable for further analysis if

- The gene ID (mapped to its Ensembl ID) was contained in the NCI60 HuEx data, the TCGA HuEx data and the TCGA RNA-seq data
- The distance between the corrected mean log expressions of the gene in the NCI60 HuEx data set and the TCGA HuEx data set was less than one (corrected maximal difference of 2-fold)
- The distance between the corrected mean log expressions of the gene in the NCI60 HuEx data set and the normalized TCGA RNA-seq data set was less than one (corrected maximal difference of 2-fold)

Of the 14,943 genes contained in all three data sets, 7799 passed the filter and showed a correlation of 0.91 (Pearson correlation, *p* < 2.2e-16) between NCI60 HuEx 1.0 ST and TCGA RNA-seq log expression values. Consequently, the filtered genes could now be used to construct a predictor for proliferation rates.

### Expression interactions enable a strong predictor for cancer proliferation

The statistical model chosen for the prediction of the NCI60 proliferation rates was a LASSO generalized linear model (Friedman et al., 2010). Here, we aimed at obtaining a predictor which would not only have good prediction properties on the training data, but would also be able to generalize to new data. Thus, all models were evaluated in a training and validation setting. In the training setting the models were trained using the entire NCI60 data set as in usual linear regression. For the validation step, in each iteration one of the 57 data points was removed from the data set, the model trained on the remaining 56 data points and the proliferation rate predicted for the omitted data point. The strategy of predicting and evaluating each data point by a model trained on all other data points is commonly known as leave-one-out cross-validation or LOOCV. Performance was evaluated across a set of five different metrics shown in Table 1.

**Table 1:** **Several performance metrics evaluated for the models shown in Figure 2**.

**mse**	**rmse**	**mae**	**mre**	***R*^2^**	**strategy**	**order**
2.00e-07	0.0004688	0.0003391	0.0163479	0.9960625	train	1st
4.49e-05	0.0067003	0.0052876	0.2611128	0.1958045	validation	1st
2.00e-07	0.0004420	0.0003570	0.0169304	0.9965005	train	2nd
8.10e-06	0.0028480	0.0022301	0.1047906	0.8547042	validation	2nd
2.00e-07	0.0004420	0.0003570	0.0169304	0.9965005	train	1st and 2nd
8.10e-06	0.0028480	0.0022301	0.1047906	0.8547042	validation	1st and 2nd
1.00e-07	0.0003543	0.0002643	0.0132028	0.9977519	train	2nd + cutoff
1.00e-06	0.0010053	0.0008111	0.0398894	0.9818972	validation	2nd + cutoff

**Figure 2:**
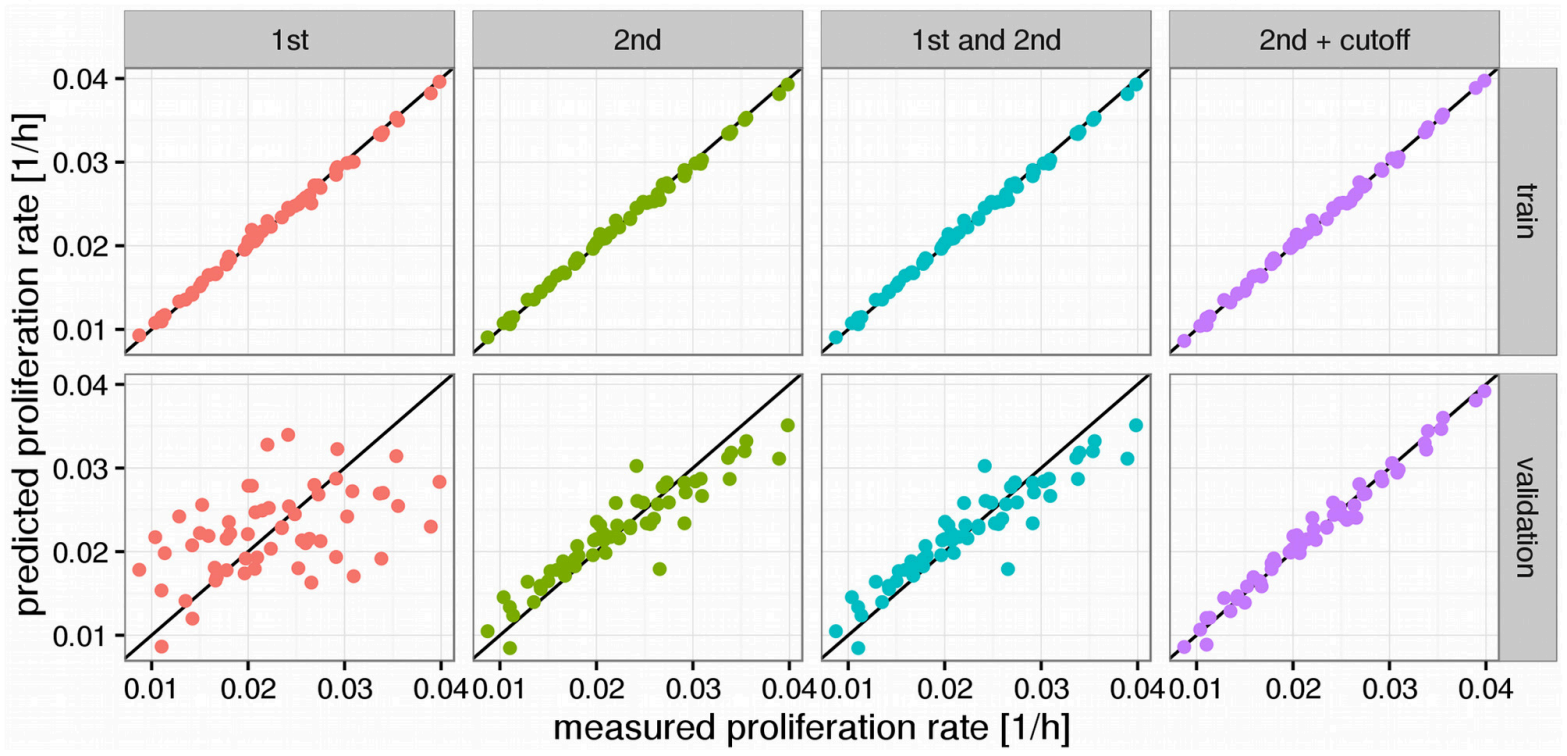
**Predictors for proliferations rates**. Panels above the figures denote the order of the model where 1st order means just the log expression values and 2nd order products between two log expression values. Black lines denote a hypothetical perfect fit (1:1 relation between measurement and prediction). “Cutoff” denotes a model where variables with very small fitted coefficients were removed from the model. Panels to the right denote the used predictions where “train” means performance on the training set and “validation” the predictions obtained from leave-one-out cross validation (LOOCV).

We observed that a simple linear model (1st order model) yielded good performance in the training step but poor performance in the validation step denoting a strong overfitting to the training data and poor generalization (see Figure 2). To alleviate this limitation we increased the order of the model by allowing for products between 1 and 2 genes as variables. This increases the computational complexity of the model training drastically since one would now have to consider more than 30 million possible combinations of the more than 7700 input genes. However, we found that it was sufficient to only consider combinations of those genes that had obtained non-zero coefficients in the 1st order case. Because merely 54 genes showed clearly non-zero regression coefficients in the 1st order model the number of tested combinations could be reduced to 1485 (1431 combinations between 2 genes and 54 squares of the individual genes). Training a pure 2nd order model with those 1485 interaction variables yielded a much stronger predictor than the first order case, particularly in the validation step where the *R*^2^ was raised from 0.2 to 0.85 compared to the 1st order model (see Figure 2, Table 1). Adding the original 1st order variables to the second order ones however did not improve the performance of the model further and we thus decided to continue with the pure 2nd order model. In a final step we tried to further improve the generalization of the predictor by removing those gene combinations with only very small regression coefficients to avoid overfitting. This was achieved by removing the 25% smallest non-zero absolute coefficient values from the model. This gave a slight improvement in the validation step to an *R*^2^ of 0.98 which now allowed stable prediction of the NCI60 proliferation rates with a relative error of 4% (Figure 2, Table 1).

**Figure 3:**
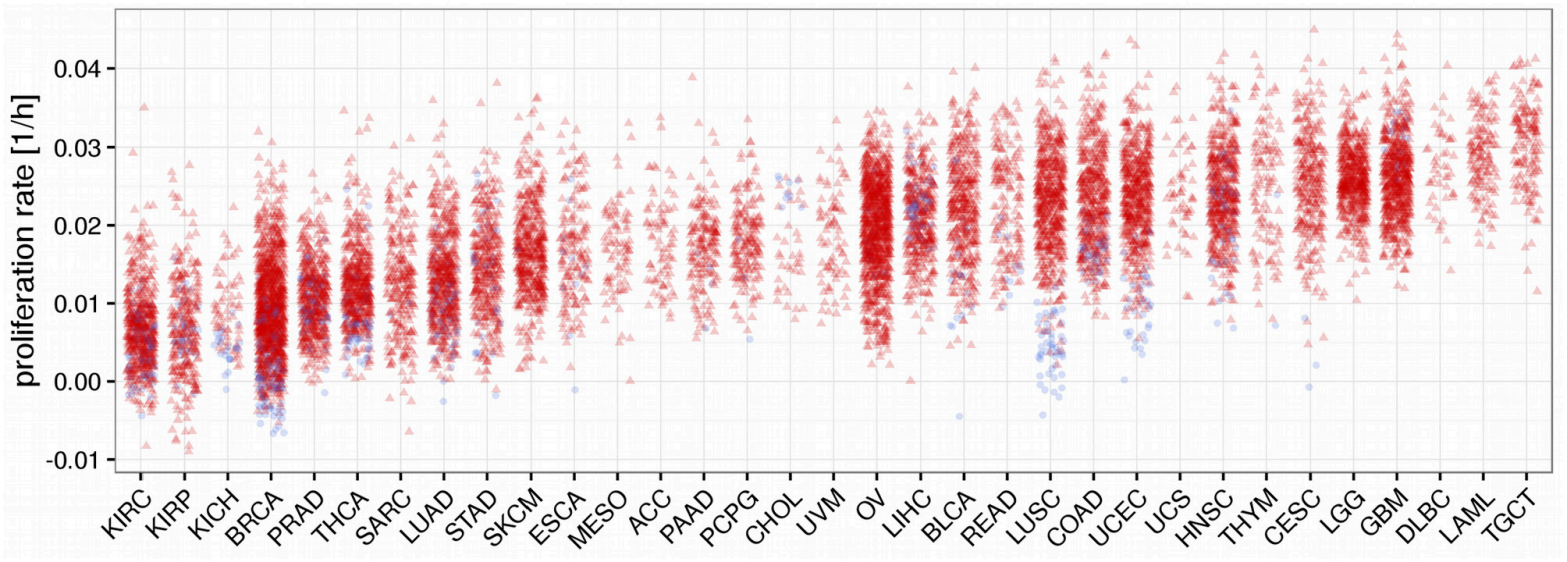
**Predicted proliferation rates across 33 cancer panels**. Cancer panels are ordered by their mean proliferation rate. Red triangles denote tumor samples and blue dots normal samples.

Using the trained model we now predicted proliferation rates for all 11,483 tumor tissue and all 756 normal tissue samples in TCGA having either associated RNA-seq or HuEx data (see Figure 3). Since the prediction is bound to make some errors it is possible that some of the proliferation rates are predicted to be negative which has no clear interpretation. In our analysis more than 98% of the predicted proliferation rates were larger than zero and negative proliferation rates were in the order of the absolute error predicted by the leave-one-out cross-validation (LOOCV 8e-3 vs. 9e-3 observed) suggesting that the negative proliferation rates actually were from samples that did not proliferate (proliferation rate is zero). As shown in Figure 3 proliferation rates were heterogeneous within and across the different cancer panels. Interestingly the separation between normal and tumor samples was only pronounced in some of the cancer panels. This is consistent with previous studies that have found large heterogeneities in proliferation rates where proliferation rates may differ even more between different cancer panels than between normal and tumor cells within the same panel (Burrell et al., 2013; Wang et al., 2013; Tomasetti and Vogelstein, 2015). For instance, the predicted proliferation rates for normal and tumor tissue samples separated well for lung squamous cell carcinomas, but not for lung adenocarcinomas.

**Figure 4:**
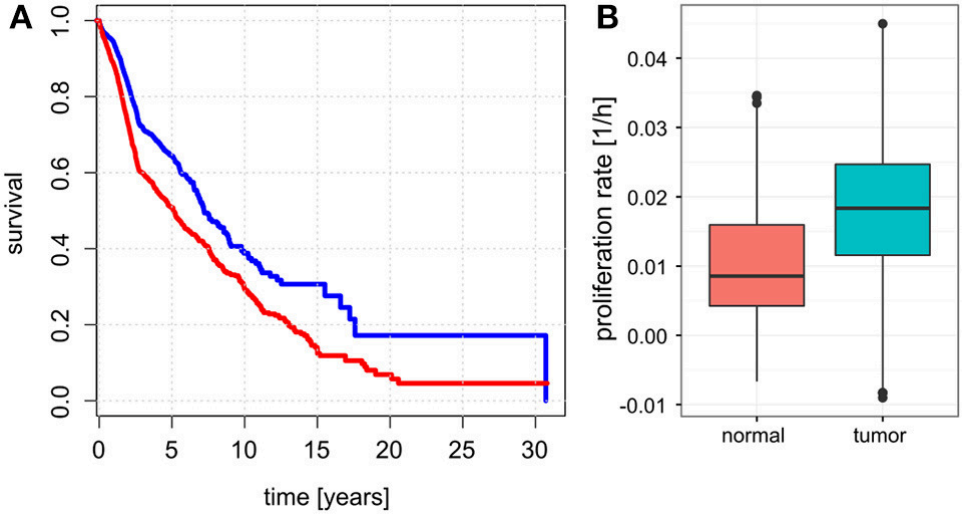
**Relationship between predicted proliferations with survival and sample type. (A)** Kaplan-Meier plots showing survival for the patients with samples falling in the lowest 25% of proliferation rates (blue) and top 25% of proliferation rates (red). **(B)** Proliferation rates between normal and tumor samples.

**Figure 5:**
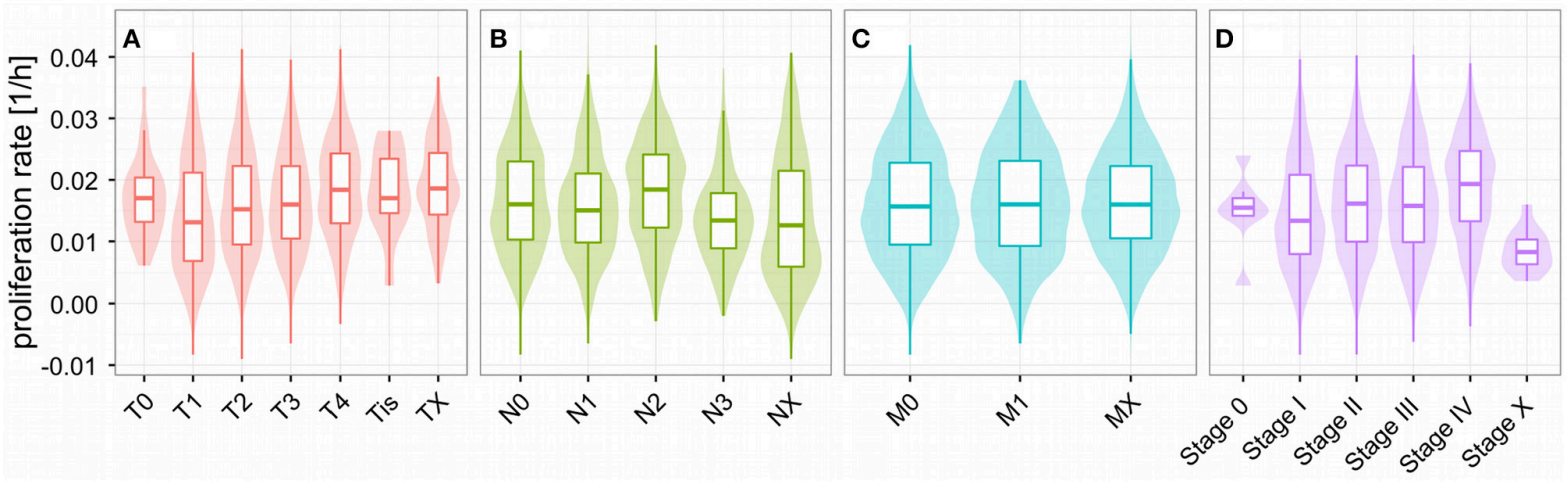
**Proliferations rates across the TNM cancer staging system**. Predicted proliferation rates across subclasses of the TNM staging system. The shaded area denotes the distribution (density) for the proliferation rates in the staging subclass. Shown are the subclasses for the size and extent of the main tumor **(A)**, number of affected lymph nodes **(B)**, distant metastasis **(C)**, and overall stage **(D)**.

Unlike for cancer cell lines, there are no reported proliferation rates across the analyzed cancer panels. Thus, we looked for alternative strategies to validate the predicted proliferation rates and studied their association with clinical data. Here, 12,111 samples had reported clinical data from 10,706 unique individuals. Comparing the Kaplan-Meier survival curves of the lower and upper quartiles of predicted proliferation rates (Figure 4A) we found a clear protective effect of lower proliferation rates on patient survival which could also be confirmed by a Cox proportional hazards model (β = 16.7, *p* < 2.2e-16). This indicates that, for instance, an increase of 0.01 in predicted proliferation rate leads to a 19% in risk. This is consistent with the expectation that more proliferative cancer should be more aggressive in general. Because cancer is mostly characterized by its ability for uncontrolled proliferation we also hypothesized that the tumor samples should show globally higher predicted proliferation rates than the normal tissue samples. This was indeed the case with tumor samples having 75% higher proliferation rates than normal tissue samples in average (Figure 4B, Wilcoxon rank sum test *p* < 2.2e-16, see Protocol S1). Finally, we also tested the association of the predicted proliferation rates with the cancer TNM staging system. Here, we found a significant association of the predicted proliferation rates with 3 of the 4 substages (Kruskal-Wallis rank sum test for T, N, stage with all p-values smaller 2.2e-16, see Protocol S1), however, this was accompanied by large variations. Proliferation rates across the subclasses of the staging system are shown in Figure 5. Predicted proliferation rates seemed to increase linearly across the T subclass between classes T1-T4 (associated with tumor size) and general tumor stage between stages I-IV (Figures 5A,D). Interestingly, subclasses such as T0, N0, or Stage 0 which are carcinomas *in situ* or tumor that were to small to be classified showed higher proliferation rates than many of the higher classes (compare for instance T0 and T1) suggesting that correct diagnosis of those small tumors is important since they might be more aggressive than tumors in the other low stages.

### Flux analysis suggests the metabolic liabilities of cancer

As mentioned earlier, one of the prevalent methods to study metabolism in cancer patients is the use of metabolic modeling and FBA. One of the usual limitations in trying to obtain the flux distribution for a specific tissue or sample is that even under knowledge of the model there is some uncertainty about the upper and lower flux bounds which may strongly influence the solution. One method to overcome this limitation is parsimonious FBA which looks for the most economic flux distribution yielding a predefined metabolic target (Lewis et al., 2010). In cancer proliferation this target can be set to be the measured or predicted proliferation rate of the cancer. Parsimonious FBA can then be used to obtain the flux distribution yielding the given proliferation rate and minimizing the sum of absolute flux values. Since this is a minimization problem it can be obtained from a model with infinitely large upper bounds and, thus, requires no knowledge about constraints in an irreversible model. Here the limiting factor is the availability of tissue reconstructions that allow for the required metabolic function (in our case proliferation). Unfortunately, many previously published reconstructions obtained by mCADRE or tINIT tissue reconstructions do not use a growth objective and are therefore not suitable for parsimonious FBA with known proliferation rates (Wang et al., 2012; Pornputtapong et al., 2015). However, there are some cancer-specific reconstructions which do allow for proliferation and have been validated qualitatively validated with experimental data (Gatto et al., 2014). Those models were reconstructed using proteome data specific for the cancer panel, thus representing the inclusion of an additional data source next to the gene expression data used to predict the proliferation rates.

**Figure 6:**
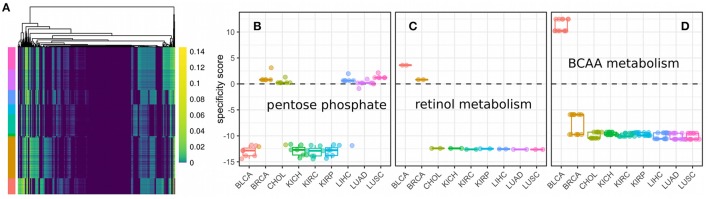
**Fluxes and metabolic specificity forn nine cancer panels. (A)** Fluxes as predicted by metabolic modeling incorporating the predicted proliferation rate across 3825 samples from nine cancer panels. Rows denote samples, columns denote non-zero fluxes (1026 in total) and colors the flux value. Cancer panels are indicated by color annotations on the rows and have the same colors and order (from top to bottom) as the panels in **(B)**. **(B)** Specificity scores for each of the fluxes of the pentose phosphate pathway. Each point denotes a single flux. A specificity score of 0 means the flux has the same value in samples within the panel as in samples outside the panel. Positive and negative values denotes a higher (or lower respectively) flux within the panel than outside the panel. **(C)** Specificity scores for each of the fluxes of retinol metabolism. **(D)** Specificity scores for each of the fluxes of branched-chain amino acid (BCAA) metabolism.

Here, we used parsimonious FBA to obtain the flux distributions for 3825 samples from nine cancer panels across unique five tissues. Fluxes were split up into their forward and reverse reaction respectively and we only considered fluxes that were non-zero in at least one sample (1026 fluxes, see Figure 6A). We observed varying usage of Glycolysis/Gluoneogenesis, Oxidative phosphorylation and the TCA cycle across the nine cancer panels (shown in Figure S1). Here, bladder cancers and breast cancers showed the highest fluxes in Glycolysis, whereas breast cancers showed diminished fluxes in the TCA cycle compared to bladder cancers. All other panels showed relatively low metabolic fluxes compared to bladder and breast cancers. Fluxes varied considerably within and across different samples (compare Figure 6A). Within a single cancer panel, this is expected since all samples in a panel used the same metabolic model constrained by the predicted proliferation rates which show strong variations as shown in Figure 3. However, the clearest pattern could be observed in the presence of absence of particular fluxes across cancer panels, indicating that the model reconstruction has more impact than the exact flux values. Direct comparison of fluxes or metabolic processes between normal and tumor conditions is difficult because of the lack of reconstructions for normal tissues with the ability to grow. Thus, we rather tried to find metabolic processes that were either regulated specifically in one cancer panel or homogeneously across all cancer panels. In order to identify pathways which were specific for a particular cancer panel we calculated a specificity score ${\text{s}}_{p}^{i}$ as the log fold-change of the mean for each flux *v*_*i*_ between the target panel and all other panels (see Data and Methods). A value of 0 marks fluxes that are homogeneous across all cancer panels, whereas a high positive or negative value denotes fluxes which are higher (or lower, respectively) in the target cancer panel. The distribution of specificity score across different metabolic pathways and cancer panels is shown in Figure S2.

**Figure 7:**
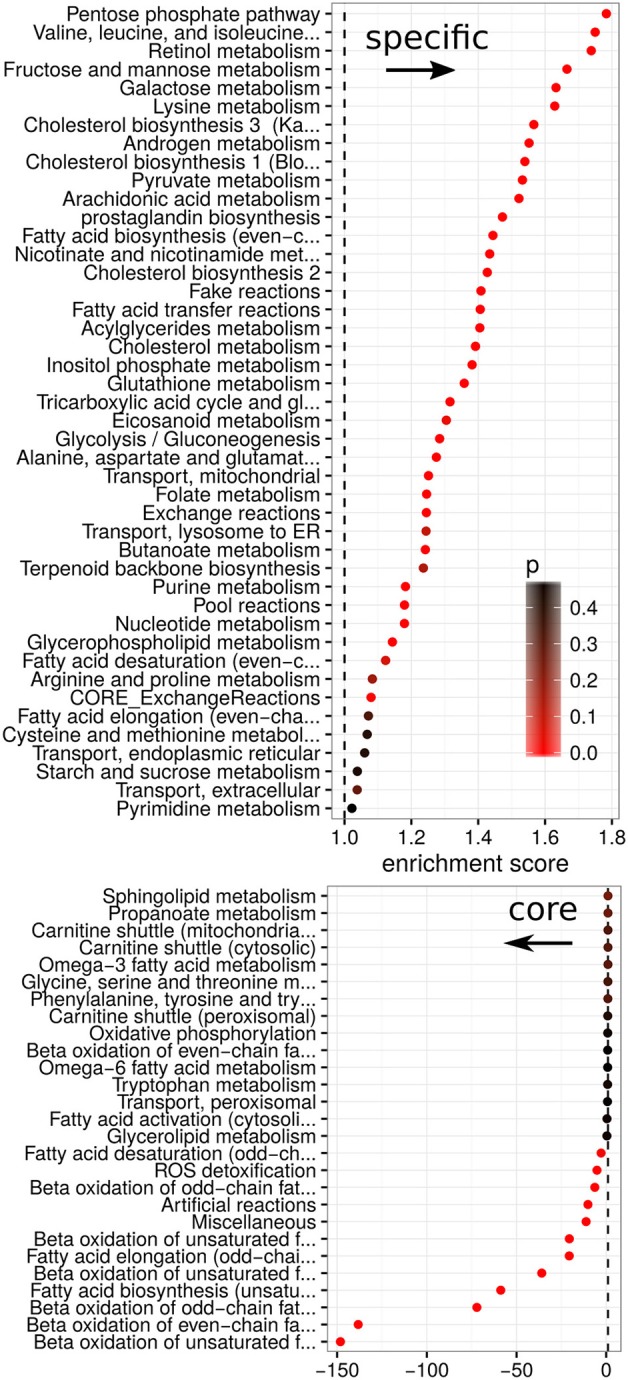
**Enrichment scores between metabolic pathways and specificity**. Enrichment for each metabolic pathway in relation to its specificity score. Scores are split into positive (enriched in specific fluxes) and negative (enriched in homogeneous fluxes). Colors denote the empirical *p*-value obtained from 100 random permutations of pathway labels.

Finally, cancer panel-specificity of the fluxes was mapped to the metabolic pathway level by calculating an enrichment score as used by GSEA (Subramanian et al., 2005) for metabolic pathways based on the specificity scores (shown in Figure 7). Here, an enrichment score of 1 denotes that the pathway is not enriched in any manner, whereas scores larger than one denotes pathways whose fluxes are specific across cancer cell panels and a score smaller than one denotes pathways which are homogeneous across panels and define a set of core pathways (see Data and Methods). The most specific pathways were the Pentose phosphate pathway, retinol metabolism and the metabolism of branched amino acids whose specificity scores are shown in Figures 6B–D. Our results suggest that pentose phosphate activity is highly heterogeneous across the studied cancer panels with metabolic fluxes being specifically up-regulated in breast cancer, cholangiocarcinoma, hepatocellular carcinoma and lung cancers (Figure 6B). The observed heterogeneity of pentose phosphate pathway activity is consistent with the literature (Cancer Genome Atlas Research Network, 2013; Du et al., 2013; Li et al., 2014; Patra and Hay, 2014; Dick and Ralser, 2015). Retinol metabolism has been shown to be altered in breast cancer and, as shown in Figure 6C, we find its fluxes specifically up-regulated in the breast and bladder cancer panel (Chen et al., 1997; Wei et al., 2015). Similarly, branched amino acid metabolism was specifically up-regulated in the bladder cancer panel (Figure 6C). Branched chain amino acid metabolism is known to be affected in cancers as well (Mayers et al., 2014; Chang et al., 2016), however, its relation to cancers is complex since it may also indicate a prior diabetic condition (O'Connell, 2013). Pathways showing homogeneous activity across the cancer cell panels all fell in the category of fatty acid metabolism-related pathways or reactive oxygen species detoxification. This is not surprising since fatty acid metabolism and oxidative stress have long been known to be involved in various cancers (Moreno-Sánchez et al., 2007; Reuter et al., 2010; Carracedo et al., 2013; Currie et al., 2013; Sosa et al., 2013; Camarda et al., 2016; Yang et al., 2016).

## Discussion

In this study we extended the gene expression profiling data contained in the Cancer Genome Atlas with predictions of proliferation rates for more than 12,000 samples. Our results suggest that the heterogeneity between and within different cancer panels is also found on the level of proliferation. Even though there is a tendency for certain cancer types to have higher proliferation rates, there is a large overlap in proliferative capabilities between different cancers. As we show the predicted proliferation rates are connected with patient survival and in differentiating normal from tumor samples and thus might be consequential for clinical investigations, particularly in early cancer stages where pathological classification is difficult.

This opens the door for more complex schemes where phenotypic traits from model systems such as cancer cell lines can be extrapolated to individual patients. However, the proliferation rate is only one of many features that determines the outcome of a particular cancer. Additionally, metabolic fluxes seem to depend more on the presence or absence of biochemical reactions than the bounds imposed by achieving a particular proliferation rate. In this analysis we used the same metabolic model for all samples of a given cancer panel. This is obviously only an approximation, albeit a recent study found sample-specific metabolic reconstructions to differ only moderately within a single cancer panel (preprint, http://dx.doi.org/10.1101/050187). There may exist many additional metabolic constraints that vary across different cancer cell samples and cancer panels such as availability of metabolites in the microenvironment, mutations of metabolic enzymes and the required metabolic capacities to resist the immune system or apoptosis. Therefore, it would be worthwhile to predict several additional phenotypic traits for the samples of The Cancer Genome Atlas. This could for instance be based on particular metabolic indicators such as the redox balance, the level of oxidative stress or the balance between the Glycolysis and the TCA cycle. As we have shown, data obtained from cell lines can be an acceptable alternative and has the potential to further constrain the solution space of metabolic modeling.

The advantages of having predictions for distinct biological phenotypes for single patient data lie in its ability to predict metabolic alterations in a more complex fashion than just analyzing the gene expression and mutations of metabolic enzymes. Particularly, it allows the inclusion of additional data through the metabolic model such as the fulfillment of metabolic requirements such as the maintenance of a viable redox balance and the uptake of the necessary nutrients from the microenvironment. As shown in Figure 4, this allows to identify the metabolic liabilities within and across cancer panels and could also be used to find metabolic alterations specifically for a single patient. Here, we found that identified metabolic liabilities were consistent with previous publications in predicting alteration in lipid metabolism as a general theme across different cancers and identifying several specific metabolic alterations in the pentose phosphate pathway, retinol metabolism, and branched chain amino acid metabolism as alterations. As more reconstructions for normal tissues become available this list is likely to be extended by comparisons between normal and tumor tissues, however that would require the inference of metabolic constraints beyond proliferation or growth rates as many normal tissues do not grow significantly. Additionally, the methodology could probably be improved by using patient-specific reconstructions for the metabolic models that better capture the inherent heterogeneity. However, that would require fast reconstruction methods in order to produce personalized models in a high-throughput fashion.

Finally, after initial model training, prediction for new samples is very efficient and can help to reduce the amount of required data. In our study we only required gene expression levels for 38 unique genes in order to predict proliferation rates with an accuracy of 96%. Additionally, all of those genes were consistently expressed across all cancer panels and cell lines and had sufficiently high expression values to be quantified reliably by RNA-Seq and microarrays. This enables cost efficient clinical probing in order to quantify phenotypic traits that can usually not be observed directly.

## Author contributions

CD developed the methods, performed the analysis and wrote the paper. OR developed the methods and wrote the paper.

## Funding

The authors thank the financial support of the Research Chair on Systems Biology (INMEGEN-FUNTEL Mexico) and from an internal grant of the National Institute of Genomic Medicine.

### Conflict of interest statement

The authors declare that the research was conducted in the absence of any commercial or financial relationships that could be construed as a potential conflict of interest.

## Supplementary material

Figure S1 **Fluxes for the major metabolic pathways across the 9 used cancer panels**. Each point denotes a single flux value for a specific sample. Shown are 141,525 individual flux values.

Figure S2 **Specificity scores across all metabolic pathways in the 1026 non-zero fluxes stratified by cancer panel**.
