# Supplementary figures and images for: Personalized Prediction of Proliferation Rates and Metabolic Liabilities in Cancer Biopsies
Source: Front Physiol. 2016 Dec 27;7:644. doi: 10.3389/fphys.2016.00644 (PMC5186797; doi:10.3389/fphys.2016.00644)

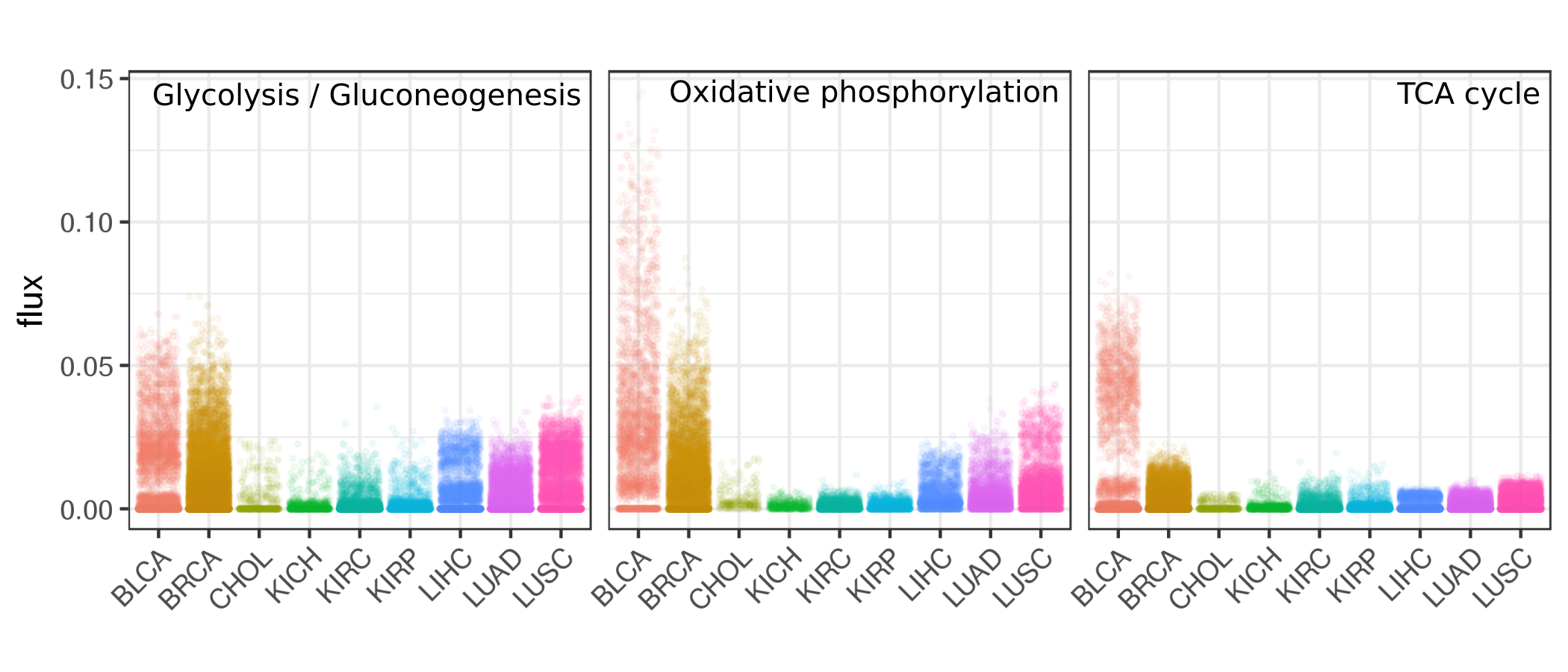

Supplement: Figure S1 — Fluxes for the major metabolic pathways across the 9 used cancer panels. Each point denotes a single flux value for a specific sample. Shown are 141,525 individual flux values. [file Image1.PNG]

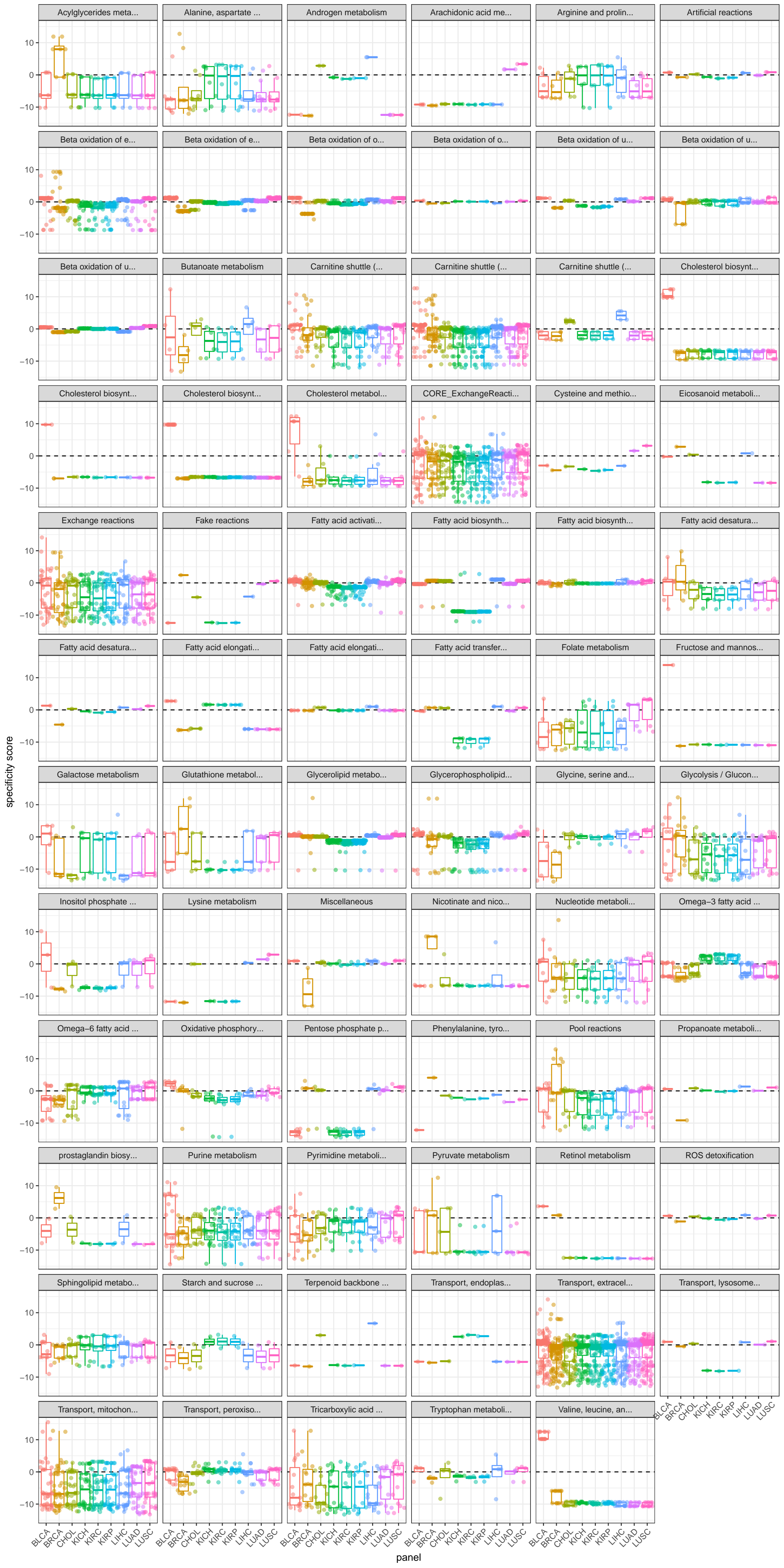

Supplement: Figure S2 — Specificity scores across all metabolic pathways in the 1026 non-zero fluxes stratified by cancer panel. [file Image2.pdf]
