# Supplementary material for: Personalized Prediction of Proliferation Rates and Metabolic Liabilities in Cancer Biopsies
Source: Front Physiol. 2016 Dec 27;7:644. doi: 10.3389/fphys.2016.00644 (PMC5186797; doi:10.3389/fphys.2016.00644)
Supplement: Supplementary file 3 [file DataSheet1.PDF]

Protocol S1

Christian Diener, Osbaldo Resendis-Antonio

Contents

1 Installation **2**

1.1 Locally . . . 2

1.2 With a cloud provider . . . 3

2 Getting the data **3**

2.1 Obtain raw data (slow) . . . 4

2.2 Obtain intermediate data (faster) . . . 4

3 Preprocessing exon expression data **4**

3.1 Normalization and summary . . . 5

3.2 Joining with proliferation data . . . 5

3.3 Exporting the regression problem . . . 6

4 Preparing TCGA data **6**

4.1 Reading the data . . . 6

4.2 Comparison to NCI-60 cell line data . . . 7

4.3 Identifying genes with conserved expression . . . 8

5 Regression and prediction **12**

5.1 Tested models . . . 12

5.2 Model selection . . . 14

5.3 Prediction of proliferation rates . . . 15

6 Proliferation rates and their association with clinical data **18**

6.1 General properties . . . 18

6.2 Survival Analysis . . . 19

6.3 Association with tumor stage . . . 21

7 Flux predictions and metabolic liabilities **22**

7.1 Flux analysis . . . 23

7.2 Cancer Panel Specificity . . . 25

7.3 Enrichment Analysis . . . 28

8 Software versions **31**
8.1 R and packages 31
8.2 Python and packages 32

1 Installation

This repository is structured to enable easy installation of the required dependencies. This is enabled by the `prtools` R package that serves two major purposes:

1. It includes auxiliary functions and tests that are used during the analysis
2. It depends on all other R packages that are used throughout the analysis. Thus, installing the `prtools` package will also ensure that all other required packages are installed.

We also provide a Docker container which has everything pre-installed and can be used to run the analysis on a server in the cloud.

This repository also includes `<step>.R` files for all steps of the analysis which you can use to run parts of the analysis in an automated manner. Additional data file are also contained in this repository. In order to obtain a local copy you can clone the repository with git.

```
git clone cdiener/proliferation
```

The recommended mode of usage is via the docker images since they guarantee correct versions for dependencies and include all intermediate data.

1.1 Locally

Note that this will require a machine with 16+ GB of RAM.

If you have docker installed you can use the steps provided in the section below “With a cloud provider”.

For a local installation you will need R (http://r-project.org), git (http://git-scm.org) and Python 3 installed (http://python.org).

On Ubuntu or Debian Stretch these can be installed via

```
sudo apt-get install r-base r-base-dev python3 python3-pip git
```

Clone the repository and enter the folder:

```
git clone https://github.com/cdiener/proliferation && cd proliferation
```

For Debian Jessie we recommend updating pip on a per-user setting in order to get a version of pip that is greater than 8.1.

```
sudo apt-get install r-base r-base-dev python3 python3-pip git
pip3 install -U pip
```

The python dependencies can be installed with

```
pip3 install lxml python-libsbml numpy scipy cobra
```

R dependencies can be installed from within R (type "R" in Terminal) with

```
install.packages("devtools")
source("https://bioconductor.org/biocLite.R")
biocLite()
```

After that you can install `prtools` simply via

```
devtools::install_github("cdiener/proliferation/prtools")
```

Loading the prtools package will then also load all additional packages we will use:

```
library(prtools)
```

If you want to download the raw analysis data you will also need the GDC data transfer tool. Installation instructions can be found here. Please make sure that the `gdc-client` executable is in your path. The docker image already includes the tool.

1.2 With a cloud provider

Using a cloud provider such as Google Cloud or Amazon AWS you can use the docker image.

1. Create a new virtual machine with more than 16 GB of RAM using the CoreOS stable image.
2. Login to the machine using SSH as described by your cloud provider.
3. Get the docker image with

```
docker pull cdiener/proliferation
```

4. Run the docker image

```
docker run -d -p 8000:8787 cdiener/proliferation
```

5. Access the machine at http://your-ip:8000 where "your-ip" is the IP of your VM or "localhost" when running docker on your own machine. You will be prompted with for login information where the user and password are "rstudio".

This will present you with an R studio interface where all additional dependencies and intermediate data are available.

For a start click the "…" symbol in the file panel on the lower right and enter "/data/proliferation". Now click on "protocol.rmd" to see or run the protocol (by clicking "knit HTML") or use any of the *.R files in the same directory.

2 Getting the data

This section describes how to obtain the raw data. Alternatively it also shows how to download intermediate data sets that cut down the analysis time significantly.

2.1 Obtain raw data (slow)

We will start by getting all the required data. You will need about 40 GB of disk space for all the downloads. This step is the longest during this analysis.

First, we will download the HuEx 1.0 ST exon expression data for the NCI-60 cell lines using the `GEOquery` package (about 9 GB).

```
GEOquery::getGEOSuppFiles("GSE29682")
untar("GSE29682/GSE29682_RAW.tar", exdir="GSE29682")
```

We follow this by downloading all relevany TCGA data from the Genomic Data Commons (GDC) as well (19 GB). For that you will need a file manifest generated from the GDC data portal. Here, we use the tcgar package which we wrote specifically for this analysis. We will save the data into the `GDC` subfolder with another subfolder for each data type. File manifests for the data used in this analysis are already provided in the `GDC` folder.

```
get_data("GDC/manigest_tcga_rnaseq", "GDC/rnaseq")
get_data("GDC/manigest_tcga_huex", "GDC/huex")
get_data("GDC/manigest_tcga_clinical", "GDC/clinical")
```

Note that this will take a long time.

Finally, we will also get annotations that relate all the genes from the NCI-60 and TCGA data sets. Here, we will employ biomart to obtain mappings between the probes on the HuEx microarrays and several Gene ID systems. This step is optional since the generated `probemap.rds` file is contained in the repository as well.

```
ensembl = useMart("ensembl",dataset="hsapiens_gene_ensembl")
attrs <- c("ensembl_gene_id", "description", "external_gene_name", "ucsc",
    "entrezgene", "affy_huex_1_0_st_v2")
probemap <- getBM(mart=ensembl, attributes=attrs)
probemap <- data.table(probemap)
names(probemap) <- c("ensgene", "description", "symbol", "ucsc", "entrez", "huex")
saveRDS(probemap, "probemap.rds")
```

2.2 Obtain intermediate data (faster)

The following intermediate data files are available online:

1. NCI-60 gene expression data and proliferation rates - http://dx.doi.org/10.5281/zenodo.61980
2. TCGA data as compressed RDS file - http://dx.doi.org/10.5281/zenodo.61982
3. probe map as compressed RDS file - *Already in the Github repository*

3 Preprocessing exon expression data

Note: This entire section is optional if you downloaded the NCI-60 gene expression file (#1). Running it will require the prior download of the NCI-60 data as described in "Obtain raw data".

3.1 Normalization and summary

We will start by preparing the HuEx exon expression data for the NCI-60 data set. Here, we will read all of the raw files, calculate the log expression values and normalize the arrays with RMA. This will take about 16 GB of RAM and take a while. If you have several cores available on your machine we recommend to use them here. To use 6 cores during computations you may use the following:

```
registerDoMC(6)
```

Finally, we will save this raw expression set to a serialized format, so we do not have to repeat this step every time. This will take about half an hour.

```
if (file.exists("eset_raw.rds")) eset <- readRDS("eset_raw.rds") else {
    celfiles <- list.celfiles(recursive=T, listGzipped=T)
    raw_data <- read.celfiles(celfiles)
    eset <- rma(raw_data, target="probeset")
    rm(raw_data)
    saveRDS(eset, file="eset_raw.rds")
}
```

The expression values we obtain this way are on the level of "probe sets", sets of spots on the microarray. However, in order to obtain expression values per gene we have to summarize those probe set expression values. For this we can use `probemap.rds` mapping that we obtained earlier. First, we will prepare the probe map to give us unique mappings between genes and probe sets. We will only consider genes that map to a probe set that is present on our arrays and we will also only use probe sets that map to a gene with a known ENSEMBL ID.

```
probemap <- readRDS("probemap.rds")
probemap <- unique(probemap, by=c("ensgene", "huex"))
probemap[, huex := as.character(huex)]
setkey(probemap, huex)
eset <- eset[rownames(eset) %in% probemap$huex, ]
probemap <- probemap[huex %in% rownames(eset) & !is.na(ensgene)]
```

We now have a mapping of probe sets to genes and vice versa. We will use that to summarize the log gene expression values into the geometric mean for each gene. For that we can use the `eset_reduce` function from the `prtools` package which does that rapidly (because it is implemented in C++).

```
eset <- eset_reduce(eset, probemap$huex, probemap$ensgene)
```

3.2 Joining with proliferation data

We now have the per-gene log expression values for each of the 178 microarrays in our data set. In order to map those arrays to their respective cell line we can use the annotations contained in `samples.csv`:

```
samples <- fread("samples.csv")
head(samples)
```

```
##       geo_id  tumor  cell_line
## 1: GSM736050 Breast     BT-549
## 2: GSM736051 Breast     HS578T
```

```
## 3: GSM736052 Breast       MCF7
## 4: GSM736053 Breast MDA-MB-231
## 5: GSM736054 Breast       T47D
## 6: GSM736055    CNS     SF-268
```

So we see that there are 2-3 reptitions for each cell line. The actual growth rates are contained in `growth_rates.csv`:

```
gcs <- fread("growth_rates.csv")
head(gcs)
```

```
##    cell_line panel_name doubling_time inoculation_density
## 1:  CCRF-CEM   Leukemia          26.7               40000
## 2:     HL-60   Leukemia          28.6               40000
## 3:     K-562   Leukemia          19.6                5000
## 4:    MOLT-4   Leukemia          27.9               30000
## 5: RPMI-8226   Leukemia          33.5               20000
## 6:        SR   Leukemia          28.7               20000
```

Now, we will reduce the individual repetitions for each cell line to its mean log expression values and relate the cell lines in the microarray samples to the ones in the growth rate data set.

```
setkey(gcs, cell_line)
cell_lines <- intersect(gcs$cell_line, samples$cell_line)
eset_summ <- sapply(cell_lines, function(cl) rowMeans(exprs(eset)[,samples$cell_line==cl]))
colnames(eset_summ) <- cell_lines
```

Right, now proliferation is given in doubling time (in hours), so we will convert those to proliferation rates and also run a quick check whether the proliferation rates are ordered the same way as the microarray samples.

```
rates <- gcs[cell_lines, log(2)/doubling_time]
names(rates) <- cell_lines
if (all(colnames(eset_summ) == names(rates))) cat("Ordering is the same :)")
```

3.3 Exporting the regression problem

Finally, we will attach the proliferation rates as an aditional columns to the expression values, thus, each row now denotes a cell line, and each column a gene, where only the last column denotes the proliferation rates. This is a common format for regression and classification problems and we will save it in csv format, so that it can be used easily in other software.

```
export <- data.table(t(eset_summ))
export[, "rates" := rates]
write.csv(export, "regprob.csv", row.names=F)
```

4 Preparing TCGA data

4.1 Reading the data

If you downloaded the intermediate data (#2) you can skip the following steps and read it directly using:

```r
tcga <- readRDS("tcga.rds")
```

After downloading the raw TCGA data from GDC, we will start by reading the TCGA data into RAM. The `tcgar` package does this efficiently. We will store the results in a list with one sublist for each technology (this will take around 20 minutes depending on your server).

```r
library(tcgar)
tcga <- list(
    rnaseq = read_rnaseq("GDC/manifest_tcga_rnaseq.tsv", "GDC/rnaseq", progress=F),
    huex = read_huex("GDC/manifest_tcga_huex.tsv", "GDC/huex", progress=F),
    clinical = read_clinical("GDC/manifest_tcga_clinical.tsv", "GDC/clinical", progress=F)
)
```

Again, we will save the output in a serialized file.

```r
saveRDS(tcga, "tcga.rds")
```

4.2 Comparison to NCI-60 cell line data

In order to compare gene expression between the TCGA RNA-Seq data and NCI-60 microarrays we will need to create a data set that contains the mean expression values for all genes that are contained in the TCGA *and* NCI-60 data. For that, we begin by calculating the mean log expression values across all samples within the TCGA and NCI-60 data (RNA-Seq abundances are measured in counts, so we have to add a pseudo count in order to obtain the log). We begin by calculating the means for the RNA-Seq and HuEx data contained in the TCGA data set.

```r
all_rnaseq <- log(rowMeans(tcga$rnaseq$counts)+1, 2)
all_rnaseq <- data.table(ensgene=tcga$rnaseq$features$ensgene,
    tcga_rnaseq=all_rnaseq)
all_huex <- data.table(symbol=tcga$huex$features$symbol,
    tcga_huex=rowMeans(tcga$huex$assay))
```

We also do the same for the NCI-60 HuEx data.

```r
nci60 <- fread("regprob.csv", header=T)
rates <- nci60$rates
nci60[, rates := NULL]
all_nci60 <- data.table(ensgene=colnames(nci60), nci60_huex=colMeans(nci60))
```

We will now join those data sets by mapping their respective IDs. `tcgar` already comes with a table mapping the genes in TCGA between different IDs, `genemap`.

```r
join <- merge(as.data.table(tcgar::genemap), all_nci60, by="ensgene")
join <- merge(join, all_huex, by="symbol")
join <- merge(join, all_rnaseq, by="ensgene")
head(join)
```

```
##              ensgene   symbol
## 1: ENSG00000000003   TSPAN6
## 2: ENSG00000000005     TNMD
```

```
## 3: ENSG00000000419     DPM1
## 4: ENSG00000000457    SCYL3
## 5: ENSG00000000460 C1orf112
## 6: ENSG00000000938      FGR
##                                                                                                   des
## 1:                                                               tetraspanin 6 [Source:HGNC Symbol;Acc:HG
## 2:                                                                 tenomodulin [Source:HGNC Symbol;Acc:HG
## 3: dolichyl-phosphate mannosyltransferase polypeptide 1, catalytic subunit [Source:HGNC Symbol;Acc:H
## 4:                                                    SCY1-like, kinase-like 3 [Source:HGNC Symbol;Acc:HG
## 5:                                           chromosome 1 open reading frame 112 [Source:HGNC Symbol;Acc:HG
## 6:                                  FGR proto-oncogene, Src family tyrosine kinase [Source:HGNC Symbol;Acc:H
##    entrez nci60_huex tcga_huex tcga_rnaseq
## 1:   7105   6.102991  9.232960   11.795649
## 2:  64102   4.281391  4.533475    6.336445
## 3:   8813   7.650315  8.091734   10.909042
## 4:  57147   5.985178  6.499165    9.908830
## 5:  55732   6.206742  5.850941    9.004820
## 6:   2268   6.113765  6.895676    9.491145
```

Sometimes, those mapping between IDs are not unique, which makes it impossible to identify unique gene expression values. We will remove those cases.

```
dupes <- join$ensgene[duplicated(join$ensgene)]
print(sum(join$ensgene %in% dupes))
```

```
## [1] 0
```

```
join <- join[!(join$ensgene %in% dupes), ]
saveRDS(join, "join.rds")
```

That gives us our joined gene expression data set.

4.3 Identifying genes with conserved expression

In order to use gene expression data from the NCI-60 data set for predictions on the TCGA data set, we first have to find a set of genes with very similar expression values between cell lines and human samples. As described in the manuscript, we consider gene expression to be conserved if the expression values are correlated, meaning that the gene expression values between the two data sets follow a linear relationship. We could estimate the slope and intercept directly from TCGA RNA-Seq and NCI-60 HuEx data, however, a more stable approach is to estimate both parameters independently from additional data we have.

4.3.1 Estimating the intercept

The intercept describes a constant difference in log expression values between the two data set. We can estimate is simply by using data obtained with the same technology between the two data sets. Since we have HuEx expression data from NCI-60 as well as the TCGA data set, the intercept can be calculated as the difference in mean between the HuEx data sets.

```
norm_int <- mean(join$nci60_huex) - mean(join$tcga_huex)
```

As we can see, using this correction both HuEx data sets relate well with each other.

```
ggplot(join, aes(x=tcga_huex, y=nci60_huex)) + geom_point(alpha=0.1, stroke=0) +
    geom_abline(intercept=norm_int, size=3/4, col="dodgerblue") +
    geom_abline(intercept=sqrt(2)+norm_int, size=3/4, col="dodgerblue", linetype="dashed") +
    geom_abline(intercept=-sqrt(2)+norm_int, size=3/4, col="dodgerblue", linetype="dashed") +
    xlab("TCGA HuEx") + ylab("NCI60 HuEx") + theme_bw()
```

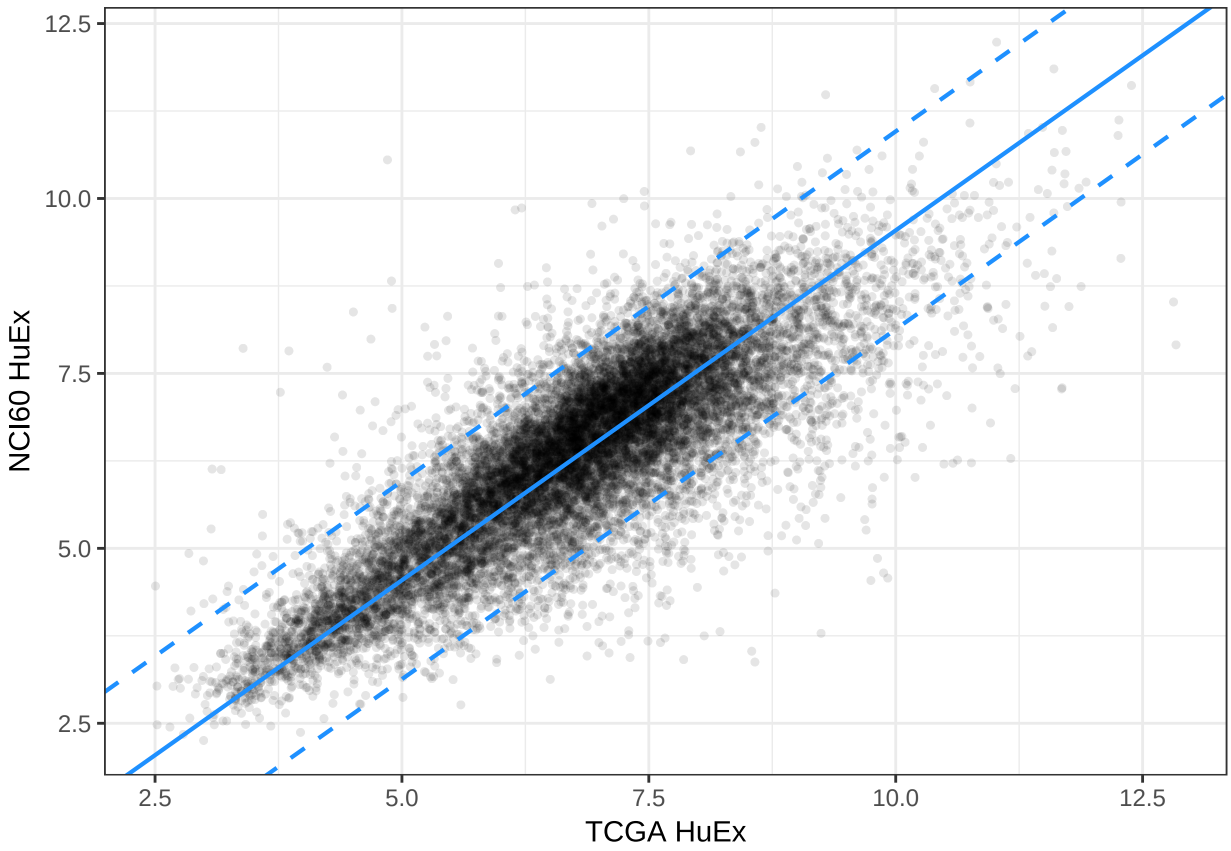

Here, the dashed blue lines denote the area in which genes have a maximum distance of 1 from the 1:1 relation denotes by the solid blue line.

4.3.2 Estimating the slope

The slope of the linear relationship denotes a difference between technologies. It can be obtained by comparing the two technologies, HuEx and RNA-Seq, within the same data set.

```
norm <- coef(glm(tcga_huex ~ tcga_rnaseq + 0, data=join))
```

Again, we see that the majority of genes follows this relationship. However, there also some genes whose expression values are not comparable, even within the TCGA data set.

```r
ggplot(join, aes(x=tcga_rnaseq, y=tcga_huex)) + geom_point(alpha=0.1, stroke=0) +
    geom_abline(slope=norm, size=3/4, col="dodgerblue") +
    xlab("TCGA RNA-seq") + ylab("TCGA HuEx") + theme_bw()
```

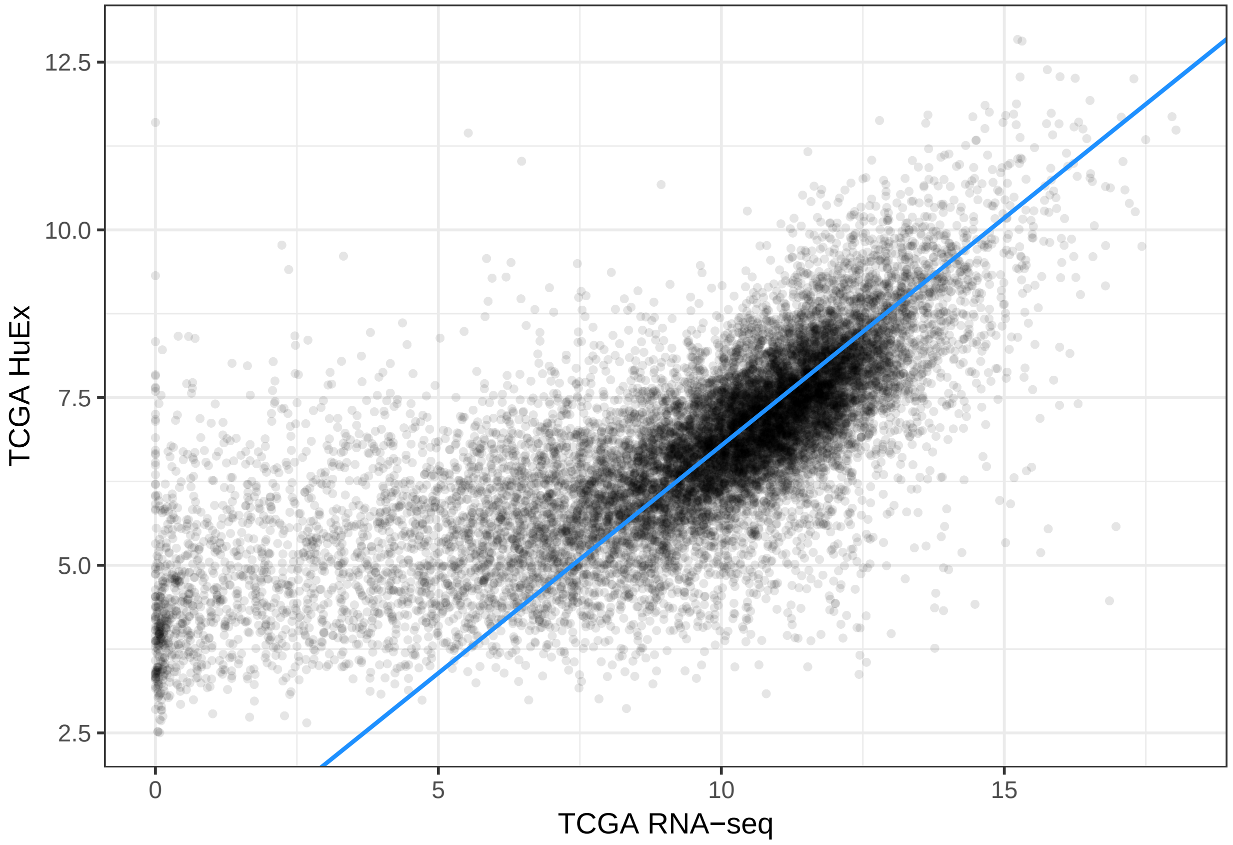

4.3.3 Validation

Finally, we can validate the two parameters by using the TCGA RNA-Seq data and the NCI-60 HuEx data.

```r
norm <- c(norm, norm_int)
saveRDS(norm, "norm_factor.rds")
ggplot(join, aes(x=tcga_rnaseq, y=nci60_huex)) + geom_point(alpha=0.1, stroke=0) +
    geom_abline(slope=norm, intercept=norm_int, size=3/4, col="dodgerblue") +
    geom_abline(slope=norm, intercept=sqrt(2)+norm_int, size=3/4,
    col="dodgerblue", linetype="dashed") +
    geom_abline(slope=norm, intercept=-sqrt(2)+norm_int, size=3/4,
    col="dodgerblue", linetype="dashed") +
    xlab("TCGA RNA-seq") + ylab("NCI60 HuEx") + theme_bw()
```

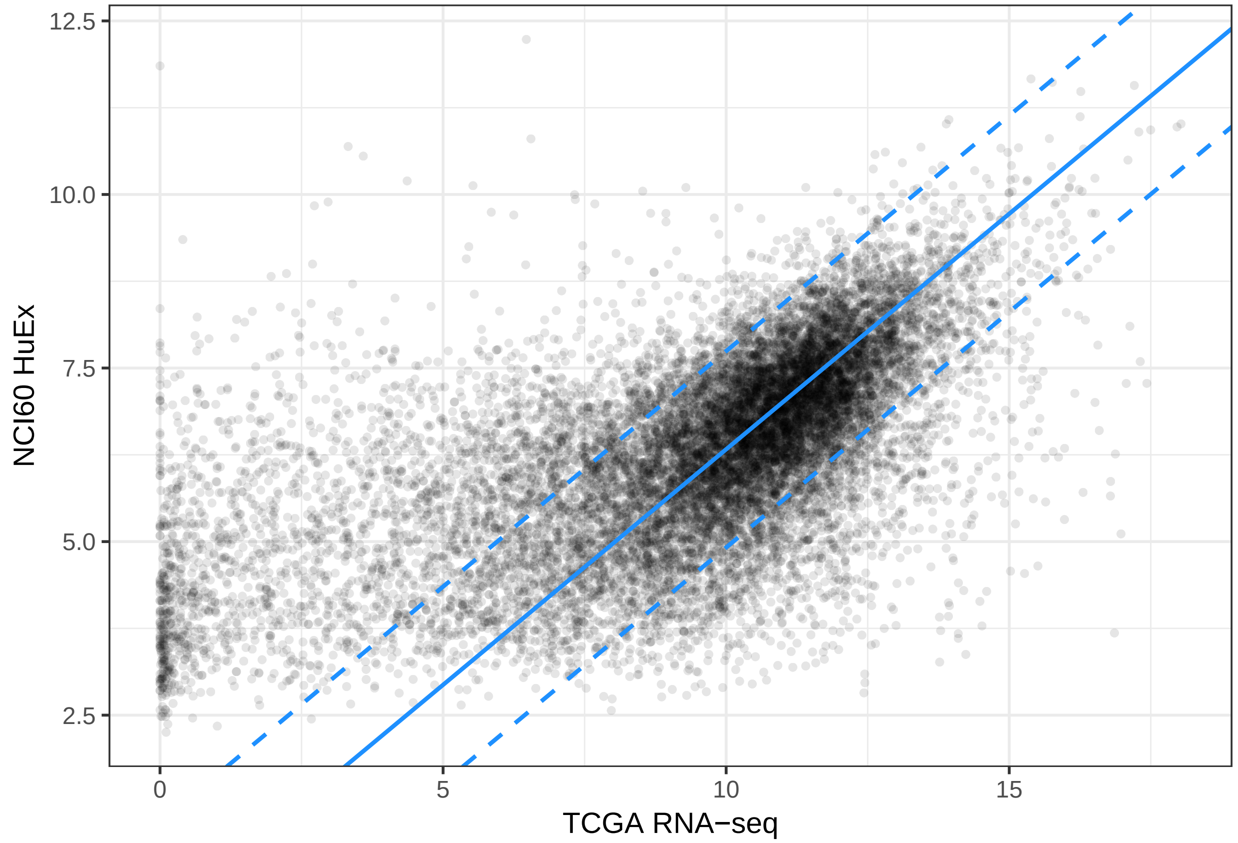

Using both parameters we see a good agreement between the data, however, there are also genes that do not follow the linear relationship. Since, we are only interested in genes with a conserved expression across the two data sets, we will only consider genes in the further analysis that do not differ by a log fold change more than 1 between the two normalized data sets (area enclosed by dashed blue lines).

```
cutoff <- 1
good <- join[(tcga_rnaseq*norm[1] + norm[2] - nci60_huex)^2 < cutoff &
    (tcga_huex + norm[2] - join$nci60_huex)^2 < cutoff, ensgene]

cor.test(join[good, tcga_rnaseq], join[good, nci60_huex])
```

```
##
##  Pearson's product-moment correlation
##
## data:  join[good, tcga_rnaseq] and join[good, nci60_huex]
## t = 197.78, df = 7797, p-value < 2.2e-16
## alternative hypothesis: true correlation is not equal to 0
## 95 percent confidence interval:
##  0.9093612 0.9167416
## sample estimates:
##       cor
## 0.9131262
```

So we see that this cutoff leads to a good general agreement.

---

5 Regression and prediction

We now have a way of normalizing the gene expression data between TCGA and NCI-60 and a criterion to select conserved genes only. Thus, we can now start with the regression problem. We start by reading the regression problem again.

```
rdata <- fread("regprob.csv", header=T)
```

But this time we will only select the genes that conserved (difference in normalized log expression smaller than one).

```
rates <- rdata$rates
rdata[, "rates" := NULL]
rdata <- as.matrix(rdata)[, good]
```

Which has dimensions

```
dim(rdata)
```

```
## [1]   57 7799
```

We will now test a total of 4 different generalized linear models.

5.1 Tested models

We will use the LASSO generalized linear models from the `glmnet` package. We will also initialize two data frames that will contain all the predictions and goodnes-of-fit metrics. Those will be calculated for each of the models on the training set and leave-one-out cross validation.

```
library(glmnet)

pred <- data.frame()
m <- data.frame()

# For leave-one-out cross validation
folds <- length(rates)
```

5.1.1 First order model

In the first model gene expression values enter as linear variables into the model. We will extract the predictions on the training set as well as predictions from leave-one-out cross validation.

```
mod1 <- cv.glmnet(rdata, rates, nfolds=folds, keep=T, parallel=T,
    grouped=FALSE, standardize=FALSE)
pred_train <- predict(mod1, rdata, s="lambda.min")[,1]
pred_test <- mod1$fit.preval[, which.min(mod1$cvm)]
```

We now append the predicted rates and performance measures to our data frames.

```
pred <- rbind(pred, data.frame(truth=rates, pred=pred_train, set="train", order="1st"))
pred <- rbind(pred, data.frame(truth=rates, pred=pred_test, set="validation", order="1st"))
m <- rbind(m, data.frame(t(measures(rates, pred_train)), set="train", order="1st"))
m <- rbind(m, data.frame(t(measures(rates, pred_test)), set="validation", order="1st"))
```

5.1.2 Second order model

In the second order model we will only consider interactions between gene expression values, meaning products between two gene expression values. Because of the large number of candidate genes it would be feasible to test all combinations. Instead we will only consider interactions between those genes that non-zero coefficients in the first order model.

```
nonzero <- abs(coef(mod1, s="lambda.min")[-1]) > 0
data2 <- inter(rdata[,nonzero])
mod2 <- cv.glmnet(data2, rates, nfolds=folds, keep=T, parallel=T,
    grouped=FALSE, standardize=FALSE)
pred_train <- predict(mod2, data2, s="lambda.min")[,1]
pred_test <- mod2$fit.preval[, which.min(mod2$cvm)]
pred <- rbind(pred, data.frame(truth=rates, pred=pred_train, set="train", order="2nd"))
pred <- rbind(pred, data.frame(truth=rates, pred=pred_test, set="validation", order="2nd"))
m <- rbind(m, data.frame(t(measures(rates, pred_train)), set="train", order="2nd"))
m <- rbind(m, data.frame(t(measures(rates, pred_test)), set="validation", order="2nd"))
```

5.1.3 First and second order model

In this model we will use the same interaction terms as before but also add all the original gene expression values in order to see whether the model can be improved.

```
data12 <- cbind(rdata[, nonzero], data2)
mod3 <- cv.glmnet(data12, rates, nfolds=folds, keep=T, parallel=T,
    grouped=FALSE, standardize=FALSE)
pred_train <- predict(mod3, data12, s="lambda.min")[,1]
pred_test <- mod3$fit.preval[, which.min(mod3$cvm)]
pred <- rbind(pred, data.frame(truth=rates, pred=pred_train, set="train", order="1st and 2nd"))
pred <- rbind(pred, data.frame(truth=rates, pred=pred_test, set="validation", order="1st and 2nd"))
m <- rbind(m, data.frame(t(measures(rates, pred_train)), set="train", order="1st and 2nd"))
m <- rbind(m, data.frame(t(measures(rates, pred_test)), set="validation", order="1st and 2nd"))
```

As we can see, adding the first order terms does not improve the second order model.

5.1.4 Regularized model by cutoff

Finally, we will try to improve the generalization of the model, by removing those interactions that have only very influences on the model and may thus be related to overfitting.

```
cf <- as.numeric(coef(mod2, s="lambda.min"))[-1]
names(cf) <- rownames(coef(mod2))[-1]
nonzero <- abs(cf) > quantile(abs(cf[abs(cf) > 0]), 0.25)
data_red <- data2[, nonzero]
mod <- cv.glmnet(data_red, rates, nfolds=folds, keep=T,
```

```
    grouped=FALSE, standardize=FALSE)
pred_train <- predict(mod, data_red, s="lambda.min")[,1]
pred_test <- mod$fit.preval[, which.min(mod$cvm)]
pred <- rbind(pred, data.frame(truth=rates, pred=pred_train, set="train", order="2nd + cutoff"))
pred <- rbind(pred, data.frame(truth=rates, pred=pred_test, set="validation", order="2nd + cutoff"))
m <- rbind(m, data.frame(t(measures(rates, pred_train)), set="train", order="2nd + cutoff"))
m <- rbind(m, data.frame(t(measures(rates, pred_test)), set="validation", order="2nd + cutoff"))
print(sum(nonzero))
```

```
## [1] 38
```

5.2 Model selection

If we compare the goodness-of-fit measures we can see that the cutoff model shows good good performance with an error of about 4% in predicting proliferation rates.

mse	rmse	mae	mre	rsq	set	order
2.00e-07	0.0004688	0.0003391	0.0163479	0.9960625	train	1st
4.49e-05	0.0067003	0.0052876	0.2611128	0.1958045	validation	1st
2.00e-07	0.0004420	0.0003570	0.0169304	0.9965005	train	2nd
8.10e-06	0.0028480	0.0022301	0.1047906	0.8547042	validation	2nd
2.00e-07	0.0004420	0.0003570	0.0169304	0.9965005	train	1st and 2nd
8.10e-06	0.0028480	0.0022301	0.1047906	0.8547042	validation	1st and 2nd
1.00e-07	0.0003543	0.0002643	0.0132028	0.9977519	train	2nd + cutoff
1.00e-06	0.0010053	0.0008111	0.0398894	0.9818972	validation	2nd + cutoff

Table 1: goodnes-of-fit measures

This can also be observed visually by plotting the real proliferation rates against the predicted ones (a perfect match would create a 1:1 line).

```
ggplot(pred, aes(x=truth, y=pred, col=order)) + geom_abline() +
    geom_point() + facet_grid(set ~ order) + theme_bw() +
    xlab("measured proliferation rate [1/h]") +
    ylab("predicted proliferation rate [1/h]") +
    theme(legend.position="none")
```

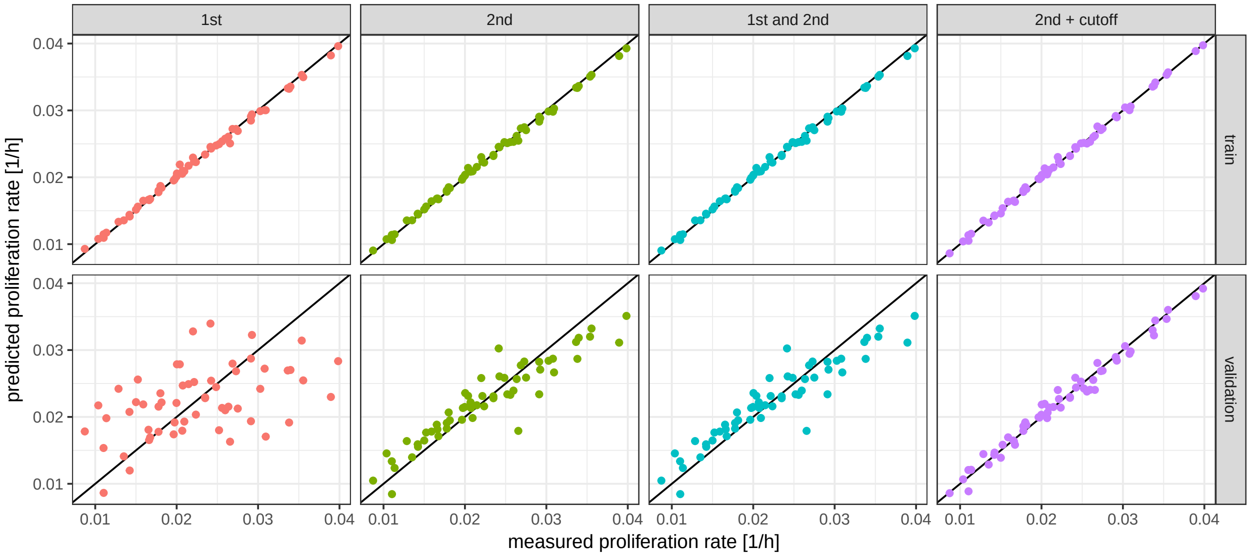

We will also save a list of the gene interactions and coefficient values for the final model.

```
genes <- do.call(rbind, strsplit(colnames(data_red), "x"))
colnames(genes) <- c("gene1", "gene2")
write.csv(data.frame(genes, coef=cf[nonzero]), "best_interactions.csv", row.names=F)
```

5.3 Prediction of proliferation rates

We now have our trained model and will continue predicting proliferation rates for the samples from TCGA.

5.3.1 Prediction for HuEx samples

The major problem we have is that in the HuEx data genes are identified by symbols whereas our interaction terms are identied by ENSEMBL gene ids. So we will begin by creating a mapping between the two using the gene map from before and getting the symbols for our genes in the interaction terms.

```
map <- genemap
setkey(map, ensgene)
symbs <- cbind(map[genes[,1], symbol], map[genes[,2], symbol])
```

With that we can get the expression values for those genes and normalize them with the normalization factor we obtained earlier.

```
huex_ex <- tcga$huex$assay[unique(as.vector(symbs)), ]
huex_ex <- huex_ex + norm[2]
```

We can now calculate the corresponding interaction terms and predict the proliferation rates for all samples. Here, we will also remove internal controls that do not map to any TCGA samples.

```
huex_red <- t(huex_ex[symbs[,1], ] * huex_ex[symbs[,2], ])
colnames(huex_red) <- paste0(symbs[,1], "x", symbs[,2])
rates_huex <- predict(mod, huex_red, s="lambda.min")[,1]
controls <- is.na(tcga$huex$samples$tumor)
rates_huex <- rates_huex[!controls]
```

5.3.2 Prediction for RNA-Seq samples

RNA-Seq samples are identified by ENSEMBL ids as well so we can advance straight away to calculating the log expression values and applying the normalization.

```
rna_ex <- tcga$rnaseq$counts[unique(as.vector(genes)), ]
rna_ex <- log(rna_ex+1, 2)
rna_ex <- rna_ex * norm[1] + norm[2]
```

With that we can calculate the interaction terms and predict the proliferation rates.

```
rna_red <- t(rna_ex[genes[,1], ] * rna_ex[genes[,2], ])
colnames(rna_red) <- paste0(genes[,1], "x", genes[,2])
rates_rna <- predict(mod, rna_red, s="lambda.min")[,1]
```

5.3.3 Combining the data sets

Finally we will first create a prediction data set that combines all proliferation rate predictions together with the sample annotations.

```
pred <- data.table(
    patient_barcode=c(tcga$rnaseq$samples$patient_barcode,
        tcga$huex$samples$patient_barcode[!controls]),
    panel=c(tcga$rnaseq$samples$panel, tcga$huex$samples$panel[!controls]),
    rates=c(rates_rna, rates_huex),
    tumor=c(tcga$rnaseq$samples$tumor, tcga$huex$samples$tumor[!controls])
    )
```

This way we can easily check what percentage of proliferation rates was predicted as negative…

```
sum(pred$rates)/nrow(pred)
```

```
## [1] 0.0176215
```

We can merge this data set with the clinical information for each patient now.

```
comb <- merge(pred, tcga$clinical, by=c("patient_barcode", "panel"))
print(comb)
```

```
##        patient_barcode panel      rates tumor
##     1:    TCGA-02-0001   GBM 0.02420641  TRUE
##     2:    TCGA-02-0003   GBM 0.03418276  TRUE
##     3:    TCGA-02-0004   GBM 0.01989495  TRUE
##     4:    TCGA-02-0006   GBM 0.02384004  TRUE
##     5:    TCGA-02-0007   GBM 0.03056712  TRUE
##    ---
## 12107:    TCGA-ZS-A9CG  LIHC 0.01406726  TRUE
## 12108:    TCGA-ZT-A8OM  THYM 0.03907078  TRUE
## 12109:    TCGA-ZU-A8S4  CHOL 0.02606463  TRUE
## 12110:    TCGA-ZU-A8S4  CHOL 0.02548018 FALSE
## 12111:    TCGA-ZX-AA5X  CESC 0.02485634  TRUE
```

```
##                             patient_uuid gender vital days_to_contact
##     1: 30a1fe5e-5b12-472c-aa86-c2db8167ab23 female  Dead             279
##     2: df3c1d61-79c1-43e9-971a-8029497ffeab   male  Dead             144
##     3: 3ac8f759-2df0-4ef5-913f-ce05b8dd8937   male  Dead             345
##     4: 98714d95-b62e-4f34-9cd1-91542da463eb female  Dead             558
##     5: fb75eb9d-05be-4e6b-a515-f39efb15e66c female  Dead             705
##    ---
## 12107: 3f6a6891-c6d5-447b-ad32-325fd46ec32b   male Alive             341
## 12108: d9816402-1073-4b19-ba16-1c7e7c00fbb3 female Alive            1398
## 12109: e76cf9a0-061e-4a7d-ba28-5a40b8da54b8   male  Dead              NA
## 12110: e76cf9a0-061e-4a7d-ba28-5a40b8da54b8   male  Dead              NA
## 12111: 4756acc0-4e96-44d4-b359-04d64dc7eb84 female Alive             119
##        days_to_death days_to_birth                        histology
##     1:           358        -16179  Untreated primary (de novo) GBM
##     2:           144        -18341  Untreated primary (de novo) GBM
##     3:           345        -21617  Untreated primary (de novo) GBM
##     4:           558        -20516  Untreated primary (de novo) GBM
##     5:           705        -14806              Treated primary GBM
##    ---
## 12107:            NA        -20100         Hepatocellular Carcinoma
## 12108:            NA        -26923                  Thymoma; Type A
## 12109:            98        -19264 Cholangiocarcinoma; intrahepatic
## 12110:            98        -19264 Cholangiocarcinoma; intrahepatic
## 12111:            NA        -23440 Cervical Squamous Cell Carcinoma
##                 tissue_site    stage  T  N  M residual_tumor
##     1:                Brain       NA NA NA NA             NA
##     2:                Brain       NA NA NA NA             NA
##     3:                Brain       NA NA NA NA             NA
##     4:                Brain       NA NA NA NA             NA
##     5:                Brain       NA NA NA NA             NA
##    ---
## 12107:                Liver Stage II T2 NX MX             R0
## 12108: Anterior Mediastinum       NA NA NA NA             NA
## 12109:            Bile duct  Stage I T1 NX MX             R0
## 12110:            Bile duct  Stage I T1 NX MX             R0
## 12111:             Cervical       NA TX NX MX             NA
##        new_tumor_events follow_ups
##     1:                2          1
##     2:                2          1
##     3:                2          1
##     4:                2          1
##     5:                2          1
##    ---
## 12107:                2          1
## 12108:                2          1
## 12109:                1          0
## 12110:                1          0
## 12111:                2          1
```

Finally we will save those data sets for later usage.

```r
saveRDS(comb, "combined.rds")
write.csv(pred, "pred_rates.csv", row.names=F)
```

6 Proliferation rates and their association with clinical data

6.1 General properties

Having the combined data set `comb` most of the following analyses are straight forward. First we can visualize the overall distribution of proliferation rates across the TCGA cancer panels.

We will start by ordering the panels by their mean proliferation rate,

```
means <- pred[, list(val=median(rates)), by=panel]
setkey(pred, panel)
pred <- pred[means[order(val), panel]]
pred[, panel := factor(panel, levels=unique(panel))]
```

followed by plotting them with `ggplot2`.

```
ggplot(pred, aes(x=panel, y=rates, color=tumor, shape=tumor)) +
    geom_jitter(alpha=0.2, height=0, size=1) + theme_bw() + theme(axis.text.x =
    element_text(angle = 45, vjust = 1, hjust=1), legend.position="none") +
    xlab("") + ylab("proliferation rate [1/h]") +
    scale_colour_manual(values=c("royalblue", "red3"))
```

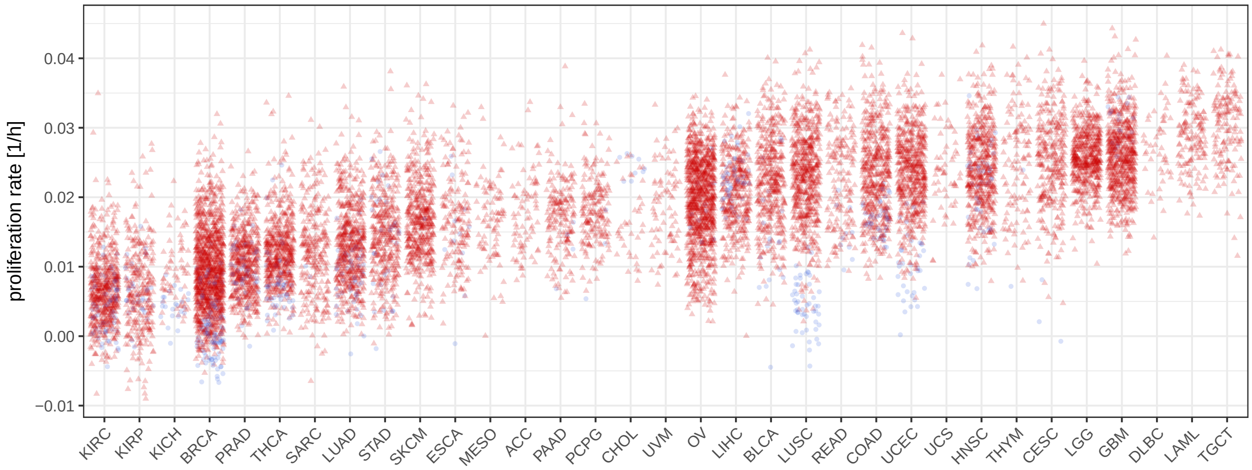


```
# Return panels to its original state
pred[, panel := as.character(panel)]
```

Here red triangles denote cancer samples and blue circles samples from healthy tissues.

We can also check how the proliferation rates from tumor and normal tissue samples behave.

```
ggplot(pred, aes(x=c("normal", "tumor")[tumor+1], y=rates, fill=tumor)) +
    geom_boxplot() + xlab("") + ylab("proliferation rate [1/h]") + theme_bw() +
    theme(legend.position="none")
```

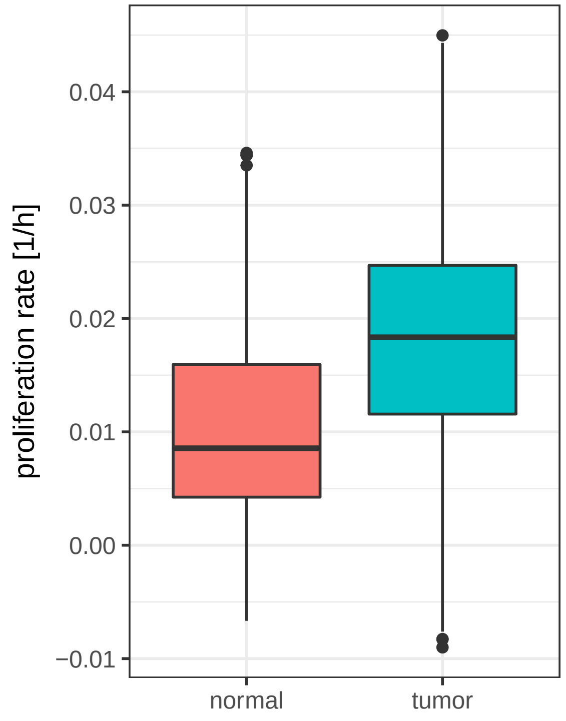

Here, the fold-change between tumor and normal tissue is given by

```
pred[(tumor), mean(rates)] / pred[!(tumor), mean(rates)]
```

```
## [1] 1.748735
```

And this difference can be tested with a wilcoxon ranked-sum test.

```
wilcox.test(pred[!(tumor), rates], pred[(tumor), rates], conf.int=TRUE)
```

```
##
##  Wilcoxon rank sum test with continuity correction
##
## data:  pred[!(tumor), rates] and pred[(tumor), rates]
## W = 2259800, p-value < 2.2e-16
## alternative hypothesis: true location shift is not equal to 0
## 95 percent confidence interval:
##  -0.008912066 -0.007526171
## sample estimates:
## difference in location
##           -0.008210097
```

6.2 Survival Analysis

First we will set up the times and censoring. The `comb` data set includes the required information in the `days_to_death`, `days_to_contact` and `vital` columns.

```
days_per_year <- 365.25

comb <- comb[!is.na(vital) & tumor]
delta <- c(comb[vital=="Alive", days_to_contact/days_per_year],
    comb[vital=="Dead", days_to_death/days_per_year])
status <- comb$vital == "Dead"
```

For visualization we will stratify the survival data into two groups. One including the top 25% of proliferation rates and the other containing the bottom 25%.

```
prolif <- vector(length=nrow(comb))
prolif[comb$rates > quantile(comb$rates, .75)] <- "high"
prolif[comb$rates < quantile(comb$rates, .25)] <- "low"
prolif <- factor(prolif, levels=c("low", "high"))
```

We can now generate the survival fit and create the corresponding Kaplan-Meier plot.

```
surv <- Surv(delta, status)
fit <- survfit(surv ~ prolif)
plot(fit, col=c("blue", "red"), xlab="time [years]", ylab="survival", lwd=2)
```

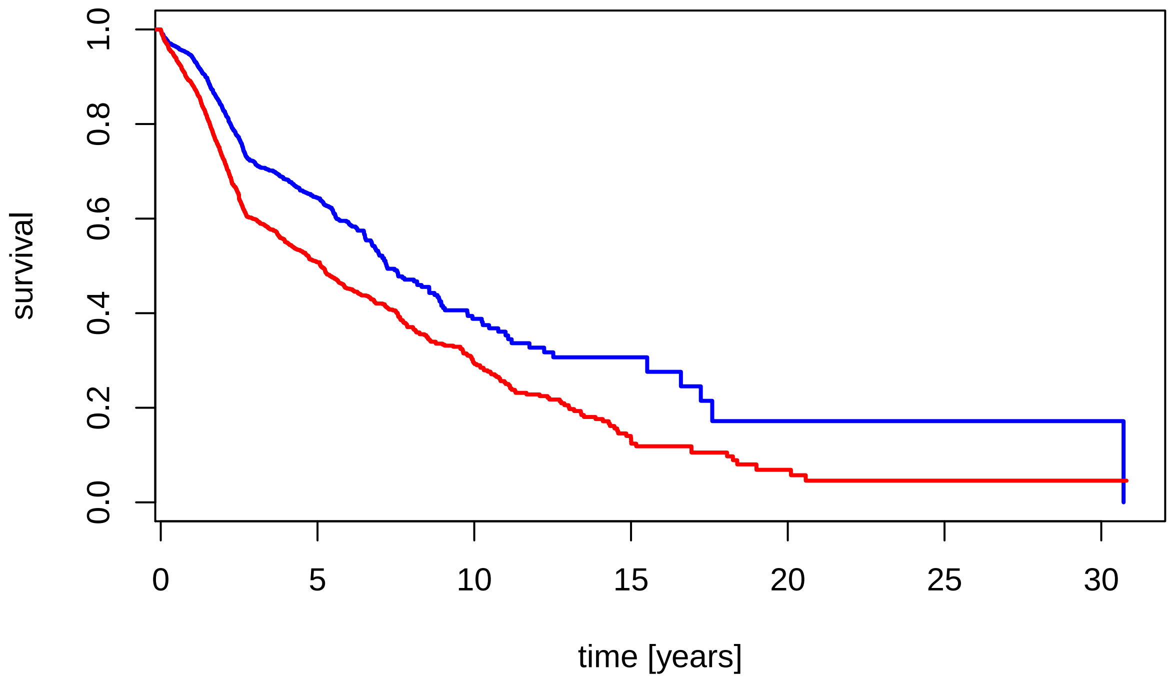

However, the proliferation rate is a continuous variable and we would like to evaluate its association to survival without creating artificial strata. For this we can use a Cox proportional hazards model.

```
coxm <- coxph(surv ~ comb$rates)
print(coxm)
```

```
## Call:
## coxph(formula = surv ~ comb$rates)
##
```

```
##                 coef exp(coef) se(coef)    z      p
## comb$rates 1.67e+01  1.75e+07 1.87e+00 8.93 <2e-16
##
## Likelihood ratio test=80.3  on 1 df, p=0
## n= 11337, number of events= 3836
##    (63 observations deleted due to missingness)
```

We can use the parameter to calculate the increase in risk when increasing the proliferation rate by a fixed value, for instance 0.01:

```
exp(coef(coxm) * 0.01)
```

```
## comb$rates
##   1.181475
```

So this would mean an increased risk of about 18%.

6.3 Association with tumor stage

We can also study how the tumor stage relates to the predicted proliferation rates. We will use the TNM staging system here. The pathological tumor stages are already contained in `comb`, however they may have several subtypes. So we will clean them up a bit to only leave the major tumor stages such as "T1", "N2", "Stage III", etc.

```
comb[, T := gsub("[a-e][0-9]*$", "", T)]
comb[, N := gsub("[^0-4NX]", "", N)]
comb[, M := gsub("[^0-4MX]", "", M)]
comb[stage %in% c("I/II NOS", "IS"), stage := NA]
comb[, stage := gsub("[A-C]*$", "", stage)]
```

This enables us to visualize the proliferation rates across the TNM staging system.

```
x <- melt(comb[, .(rates, panel, T, N, M, stage)], id.vars=c("rates", "panel"))
ggplot(x, aes(x=value, y=rates, col=variable)) +
    geom_violin(aes(fill=variable), scale="width", alpha=0.3, linetype=0) +
    geom_boxplot(outlier.colour=NA, width=0.5) +
    facet_wrap(~ variable, scales="free_x", nrow=1) + theme_bw() +
    theme_bw() + theme(axis.text.x = element_text(angle = 45, vjust = 1,
    hjust=1), legend.position="none", strip.text = element_blank()) +
    ylab("proliferation rate [1/h]") + xlab("")
```

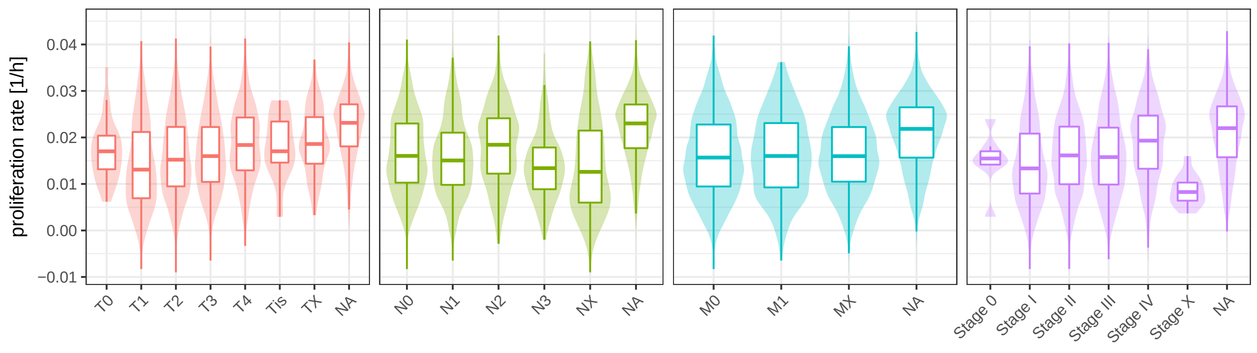

As we can see there is again a lot of heterogeneity going on. We can test for associations by the non-parametric Kruskal-Wallis test.

```
kw_tests <- x[, kruskal.test(rates, factor(value)), by=variable]
print(kw_tests)
```

```
##    variable  statistic parameter      p.value                       method
## 1:        T 194.685979         6 2.564358e-39 Kruskal-Wallis rank sum test
## 2:        N 147.075919         4 8.614847e-31 Kruskal-Wallis rank sum test
## 3:        M   1.720631         2 4.230285e-01 Kruskal-Wallis rank sum test
## 4:    stage 184.017226         5 7.418813e-38 Kruskal-Wallis rank sum test
##                   data.name
## 1: rates and factor(value)
## 2: rates and factor(value)
## 3: rates and factor(value)
## 4: rates and factor(value)
```

7 Flux predictions and metabolic liabilities

In order to predict fluxes based on the particular predicted proliferation rate and cancer panel we need a specific metabolic model for the cancer that include an objective for proliferation (growth). There are only few reconstructions that fulfill that requirement. Here, we use reconstruction from the Metabolic Atlas for 9 of the TCGA cancer panels which have been validated before (http://dx.doi.org/10.1073/pnas.1319196111). The mapping between panels and cancer models is described in `tissues.csv`:

```
tissues <- fread("tissues.csv")
print(tissues)
```

```
##                                      name panel     tissue
## 1:          Bladder Urothelial Carcinoma  BLCA urothelial
## 2:             Breast invasive carcinoma  BRCA     breast
## 3:                    Cholangiocarcinoma  CHOL      liver
## 4:                     Kidney Chromophobe  KICH     kidney
## 5:     Kidney renal clear cell carcinoma  KIRC     kidney
## 6: Kidney renal papillary cell carcinoma  KIRP     kidney
## 7:        Liver hepatocellular carcinoma  LIHC      liver
## 8:                   Lung adenocarcinoma  LUAD       lung
## 9:          Lung squamous cell carcinoma  LUSC       lung
##
## 1: http://www.metabolicatlas.org/assets/initcan/r3/urothelial_cancer_-__3.xml-b391ad03203294d3a42000
## 2:     http://www.metabolicatlas.org/assets/initcan/r3/breast_cancer_-__3.xml-4297bbc8636e6b22818d18
## 3:      http://www.metabolicatlas.org/assets/initcan/r3/liver_cancer_-__3.xml-07bff95988866a0e32afc4
## 4:      http://www.metabolicatlas.org/assets/initcan/r3/renal_cancer_-__3.xml-bb999a8ccff9ebd687264b
## 5:      http://www.metabolicatlas.org/assets/initcan/r3/renal_cancer_-__3.xml-bb999a8ccff9ebd687264b
## 6:      http://www.metabolicatlas.org/assets/initcan/r3/renal_cancer_-__3.xml-bb999a8ccff9ebd687264b
## 7:      http://www.metabolicatlas.org/assets/initcan/r3/liver_cancer_-__3.xml-07bff95988866a0e32afc4
## 8:       http://www.metabolicatlas.org/assets/initcan/r3/lung_cancer_-__3.xml-bf4255bf7e786330b1e662
## 9:       http://www.metabolicatlas.org/assets/initcan/r3/lung_cancer_-__3.xml-bf4255bf7e786330b1e662
```

For each cancer panel we will now use the corresponding model and solve the linear programming problem posed by parsimonious FBA for every cancer sample in the panel with a non-zero proliferation rate. Fluxes

are denoted by v_i, the stochiometric matrix of the model by $\mathbf{S}$, the proliferation objective in the model by v_p and the predicted proliferation rate by r_p. Models are converted to the irreversible formulation beforehand.

$$\begin{aligned} \min_i \sum_i v_i \\ s.t. \quad & \mathbf{S}\mathbf{v} = 0 \\ & v_p = r_p \\ & v_i \geq 0 \end{aligned}$$

For that we use cobrapy and warm start strategy where a previous solution basis is recycled to yield faster convergence of the solver. All of this is implemented in the `fluxes.py` script which will use the predicted rates saved earlier.

```
system2("python3", "fluxes.py")
```

Note that you may have to substitute `python3` with `python` depending on your Python installation.

7.1 Flux analysis

The resulting fluxes are now saved in `fluxes.csv`, for convenience this only includes fluxes which were non-zero in at least one sample.

First we will create a mapping between patient barcodes and cancer panels.

```
panels <- pred$panel
names(panels) <- pred$patient_barcode
panels <- sort(panels)
```

Now we can read the fluxes and conver them to a matrix.

```
fluxes <- fread("fluxes.csv")
barcodes <- fluxes$V1
fluxes <- as.matrix(fluxes[, V1 := NULL])
rownames(fluxes) <- barcodes
```

For later analysis we would also like to group fluxes by their corresponding metabolic pathways. The `fluxes.py` script also extracts that information from the model and saves is as "subsystem" in the `flux_info.csv` file.

```
info <- fread("flux_info.csv")
names(info) <- c("reaction", "subsystem")
head(info)
```

```
##              reaction                 subsystem
## 1: CancerBiomass_Ex      Artificial reactions
## 2: CancerBiomass_OF      Artificial reactions
## 3:          HMR_0002 Acylglycerides metabolism
## 4:          HMR_0003 Acylglycerides metabolism
## 5:          HMR_0004  Transport, extracellular
## 6:          HMR_0005 Acylglycerides metabolism
```

We can visulaize the resulting fluxes with a heatmap.

```r
cols <- viridis::viridis(256)
panels <- panels[!duplicated(names(panels))]
fluxes <- fluxes[order(panels[rownames(fluxes)], decreasing=TRUE), ]
in_fluxes <- names(panels) %in% rownames(fluxes)
annrow <- data.frame(panel=panels[in_fluxes], row.names=names(panels)[in_fluxes])
anncolors <- scales::hue_pal()(9)
names(anncolors) <- levels(annrow$panel)
anncolors <- list(panel=anncolors)

s <- seq(-16, log(max(fluxes)+1e-16,2), length.out = 256)
pheatmap(fluxes, breaks=c(-1e-6,2^s), col=cols, show_rownames=F,
    show_colnames=F, annotation_row=annrow, annotation_colors=anncolors,
    cluster_rows=FALSE, border_color=NA)
```

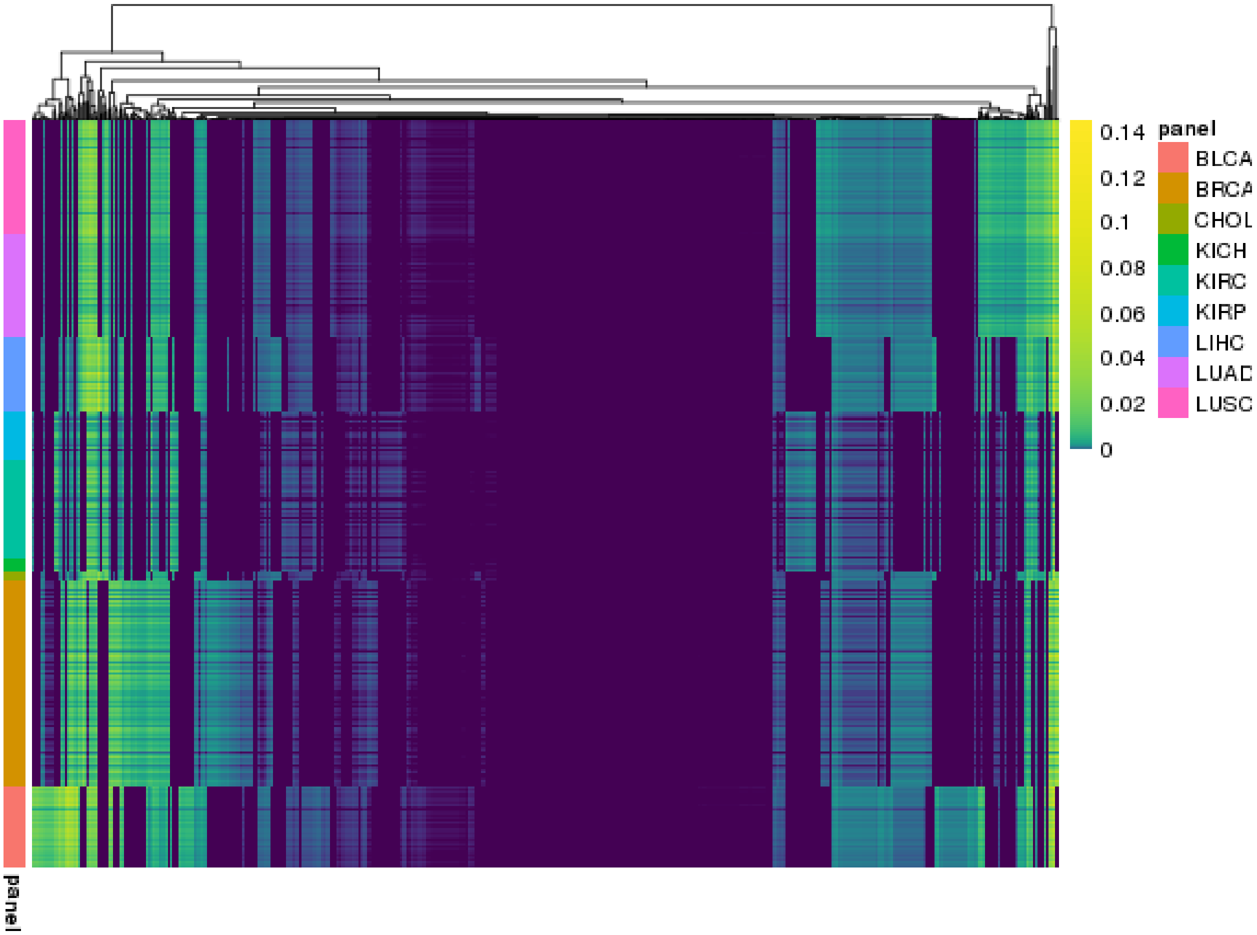

In order to see major metabolic proerties of the studied panels we will also visualize the fluxes across the three major pathways of central carbon metabolism. We will start by transforming our flux matrix to a data.table that can be passed to ggplot.

```r
dt <- as.data.table(fluxes)
dt$panel <- panels[rownames(fluxes)]
dt <- melt(dt, id.vars = "panel")
names(dt) <- c("panel", "reaction", "flux")
dt <- merge(dt, info, by = "reaction")
```

This will be followed by filtering the subsystem for Glycolysis, Oxidative Phosphorylation, and TCA cycle and plotting the results.

```
pathways <- c("Glycolysis / Gluconeogenesis", "Oxidative phosphorylation",
    "Tricarboxylic acid cycle and glyoxylate/dicarboxylate metabolism")
ggplot(dt[subsystem %in% pathways], aes(x = panel, y = flux, color = panel)) +
    geom_jitter(height = 0, alpha = 0.05, size = 0.5) + facet_wrap(~ subsystem) +
    theme_bw() + theme(axis.text.x = element_text(angle = 45, vjust = 1,
    hjust = 1), legend.position = "none") + xlab("")
```

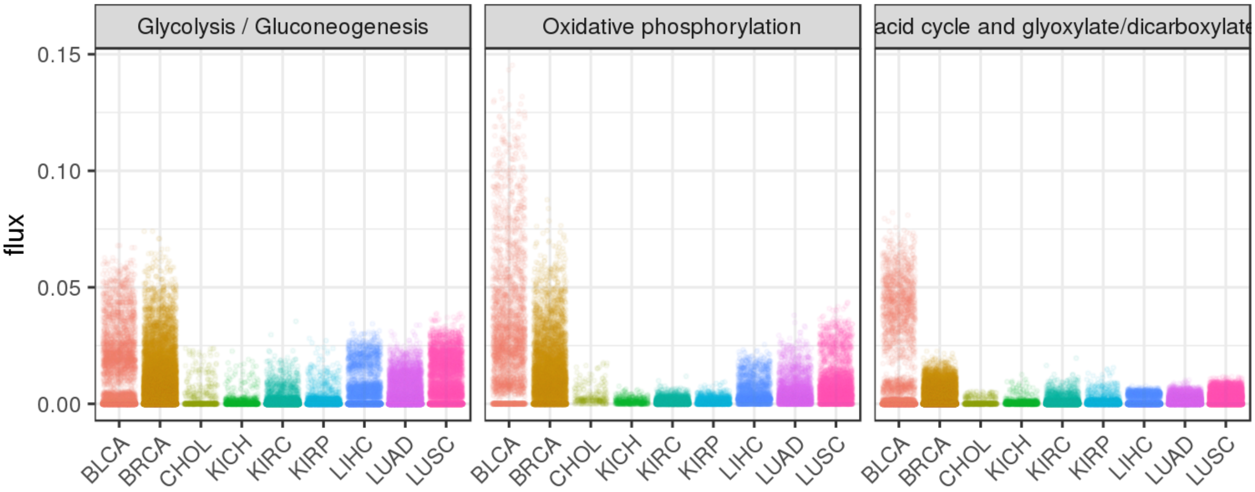

7.2 Cancer Panel Specificity

Based on the predicted fluxes we can now calculate a specificity score s_p^i for each flux v_i and panel p as the log-fold change of the flux within the panel versus all other panels. Here μ_p^i denotes the mean value of the flux v_i within the panel p and μ_o^i outside the panel p.

$$s_p^i = \log_2 \mu_p^i - \log_2 \mu_p^i$$

A value of zero denotes homogeneous fluxes across all panels, a value larger than zero fluxes that are up-regulated in a particular panel and a value smaller zero fluxes that are down-regulated in a particular panel. The calculation is implemented in the `prtools` package.

```
lfcs <- panel_lfc(fluxes, panels, info[, list(subsystem)])
```

We will order those by their absolute value from largest to smallest.

```
lfcs <- lfcs[order(-abs(lfc))]
lfcs
```

```
##                       rid panel           lfc
##    1:            HMR_4855  BLCA  1.544089e+01
##    2:            HMR_4354  KIRC -1.445816e+01
##    3:            HMR_4663  KIRC -1.443521e+01
##    4: newExRxn_39_reverse  KIRC -1.443521e+01
##    5:         newExRxn_41  KIRC -1.443521e+01
```

```
##   ---
## 9239:    HMR_2697_reverse  CHOL -4.838653e-03
## 9240:            HMR_3414  CHOL -4.838653e-03
## 9241:            HMR_4874  CHOL -3.940913e-03
## 9242:            HMR_3984  LIHC  3.425966e-05
## 9243:            HMR_4900  LIHC  3.425966e-05
##                                                         subsystem
##    1:                                Transport, mitochondrial
##    2:                               Pentose phosphate pathway
##    3:                                       Purine metabolism
##    4:                                  CORE_ExchangeReactions
##    5:                                  CORE_ExchangeReactions
##   ---
## 9239:                       Carnitine shuttle (mitochondrial)
## 9240: Beta oxidation of even-chain fatty acids (mitochondrial)
## 9241:                                Transport, mitochondrial
## 9242:                                           Miscellaneous
## 9243:                                  Transport, peroxisomal
```

We can now visualize those changes across all metabolic pathways.

```
lfcs[, panel := factor(panel)]
lab <- function(x) lapply(x, shorten, n=20)
lfc_plot <- ggplot(lfcs, aes(x=panel, y=lfc, col=panel)) +
    geom_hline(yintercept=0, linetype="dashed") +
    geom_boxplot(outlier.colour=NA) + geom_jitter(alpha=0.5) +
    theme_bw() + theme(axis.text.x=element_text(angle = 45, vjust = 1, hjust=1),
    legend.position="none") + facet_wrap(~subsystem, labeller=lab, nrow=12, ncol=6) +
    ylab("specificity score")

print(lfc_plot)
```

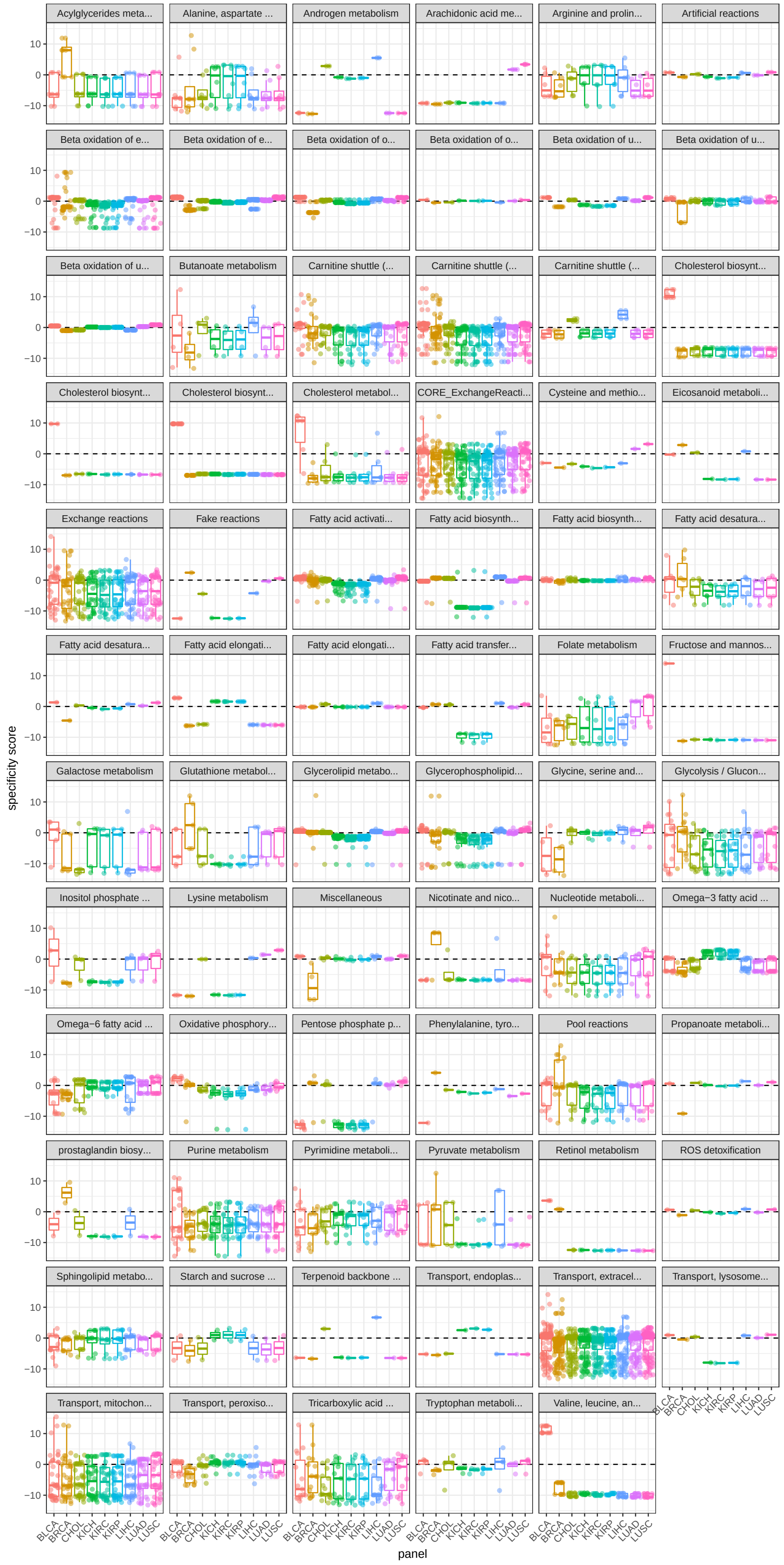
Acylglycerides meta...
Alanine, aspartate ...
Androgen metabolism
Arachidonic acid me...
Arginine and prolin...
Artificial reactions
Beta oxidation of e...
Beta oxidation of e...
Beta oxidation of o...
Beta oxidation of o...
Beta oxidation of u...
Beta oxidation of u...
Beta oxidation of u...
Butanoate metabolism
Carnitine shuttle (...
Carnitine shuttle (...
Carnitine shuttle (...
Cholesterol biosynt...
Cholesterol biosynt...
Cholesterol biosynt...
Cholesterol metabol...
CORE_ExchangeReacti...
Cysteine and methio...
Eicosanoid metaboli...
Exchange reactions
Fake reactions
Fatty acid activati...
Fatty acid biosynth...
Fatty acid biosynth...
Fatty acid desatura...
Fatty acid desatura...
Fatty acid elongati...
Fatty acid elongati...
Fatty acid transfer...
Folate metabolism
Fructose and mannos...
Galactose metabolism
Glutathione metabol...
Glycerolipid metabo...
Glycerophospholipid...
Glycine, serine and...
Glycolysis / Glucon...
Inositol phosphate ...
Lysine metabolism
Miscellaneous
Nicotinate and nico...
Nucleotide metaboli...
Omega−3 fatty acid ...
Omega−6 fatty acid ...
Oxidative phosphory...
Pentose phosphate p...
Phenylalanine, tyro...
Pool reactions
Propanoate metaboli...
prostaglandin biosy...
Purine metabolism
Pyrimidine metaboli...
Pyruvate metabolism
Retinol metabolism
ROS detoxification
Sphingolipid metabo...
Starch and sucrose ...
Terpenoid backbone ...
Transport, endoplas...
Transport, extracel...
Transport, lysosome...
Transport, mitochon...
Transport, peroxiso...
Tricarboxylic acid ...
Tryptophan metaboli...
Valine, leucine, an...
specificity score
10
0
−10
BLCA
BRCA
CHOL
KICH
KIRC
KIRP
LIHC
LUAD
LUSC
panel

7.3 Enrichment Analysis

Those are a lot of plot and we would like a more quantitative evaluation which of the pathways are the most specific or or homogeneous ones. This can be obtained from an enrichment analysis. Here we use a method similar to GSEA which calculates normalized enrichment scores (NES) and p-values for each pathway.

Here, a value larger than one denotes association with specific fluxes and a value smaller than one association with homogeneous fluxes.

```
pws <- lfcs$subsystem
enr <- sapply(unique(pws), NES, w=lfcs$lfc, pws=pws)
enr <- data.table(subsystem=colnames(enr), nes=enr[1,], p=enr[2,])
enr <- enr[order(nes)]
enr[, subsystem := factor(subsystem, levels=subsystem)]
```

For panel-specific pathways we now obtain the following:

```
enr_plot <- ggplot(enr, aes(x=nes, y=subsystem, col=p)) +
    geom_vline(xintercept=1, linetype="dashed") +
    geom_point() + scale_colour_continuous(low="red", high="black") +
    theme_bw() + scale_y_discrete(labels=shorten) +
    theme(legend.position=c(0.2,0.8)) + xlab("enrichment score") + ylab("")

print(enr_plot %+% enr[nes > 1])
```

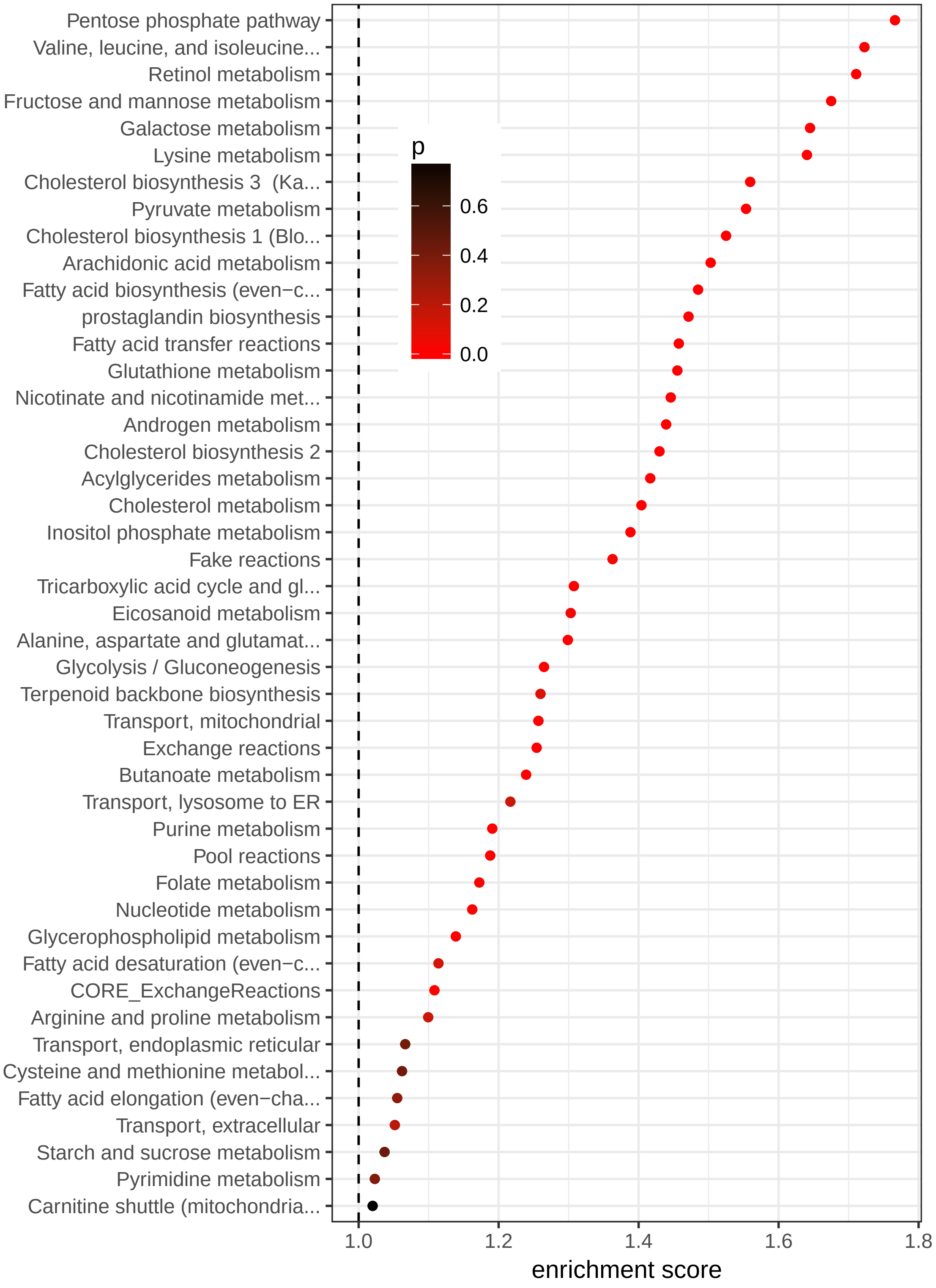

Here we see that there is a group of three pathways that set themselves apart on the top. We can visualize only those again:

```
best <- rev(enr$subsystem)[1:3]
print(lfc_plot %+% lfcs[subsystem %in% best])
```

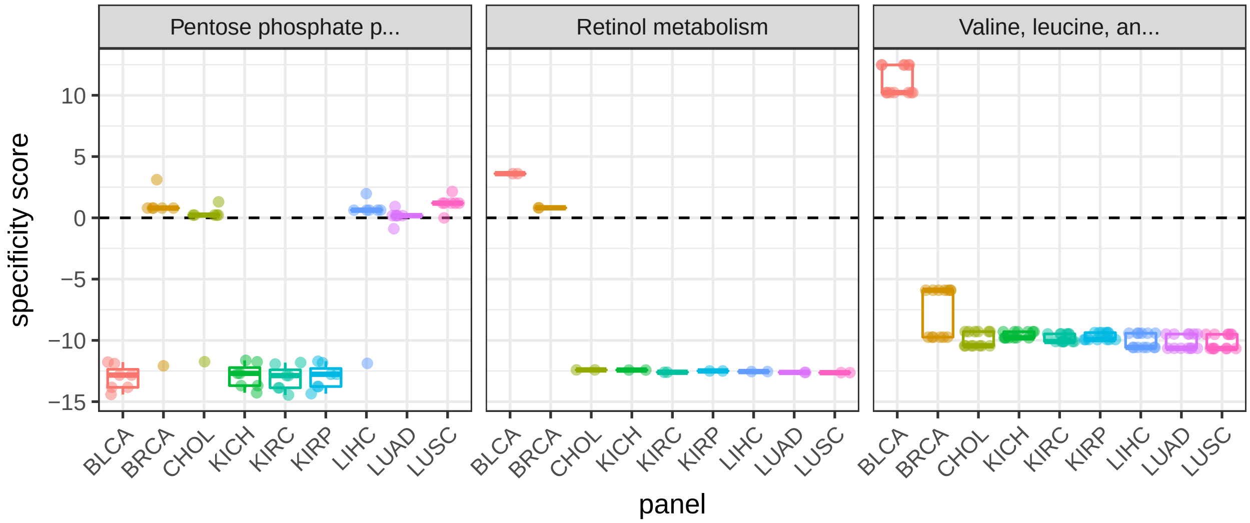

Finally we can also visualize homogeneous pathways.

```
print(enr_plot %+% enr[nes <= 1])
```

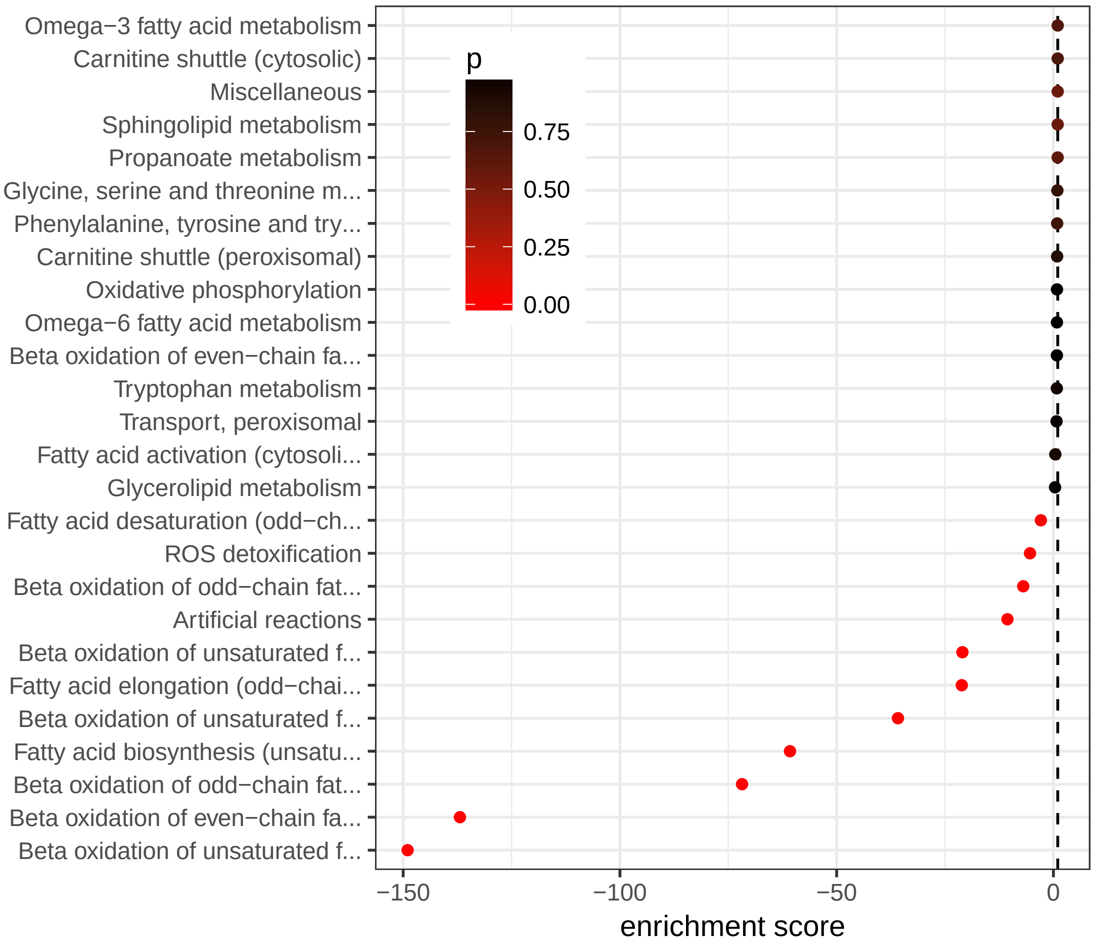

8 Software versions

8.1 R and packages

```r
sessionInfo()
```

```
## R version 3.3.2 (2016-10-31)
## Platform: x86_64-pc-linux-gnu (64-bit)
## Running under: Debian GNU/Linux 8 (jessie)
##
## locale:
##  [1] LC_CTYPE=en_US.utf8       LC_NUMERIC=C
##  [3] LC_TIME=en_US.utf8        LC_COLLATE=en_US.utf8
##  [5] LC_MONETARY=en_US.utf8    LC_MESSAGES=en_US.utf8
##  [7] LC_PAPER=en_US.utf8       LC_NAME=C
##  [9] LC_ADDRESS=C              LC_TELEPHONE=C
## [11] LC_MEASUREMENT=en_US.utf8 LC_IDENTIFICATION=C
##
## attached base packages:
## [1] stats4    parallel  methods   stats     graphics  grDevices utils
## [8] datasets  base
##
## other attached packages:
##  [1] tcgar_0.3.0         survival_2.40-1     pheatmap_1.0.8
##  [4] oligo_1.38.0        Biostrings_2.42.0   XVector_0.14.0
##  [7] IRanges_2.8.1       S4Vectors_0.12.0    Biobase_2.34.0
## [10] oligoClasses_1.36.0 BiocGenerics_0.20.0 glmnet_2.0-5
## [13] Matrix_1.2-7.1      ggplot2_2.2.0       doMC_1.3.4
## [16] iterators_1.0.8     foreach_1.4.3       data.table_1.9.6
## [19] biomaRt_2.30.0      prtools_0.3.2       RcppProgress_0.2.1
##
## loaded via a namespace (and not attached):
##  [1] Rcpp_0.12.7                lattice_0.20-34
##  [3] assertthat_0.1             digest_0.6.10
##  [5] GenomeInfoDb_1.10.1        plyr_1.8.4
##  [7] chron_2.3-47               RSQLite_1.0.0
##  [9] evaluate_0.10              highr_0.6
## [11] BiocInstaller_1.24.0       zlibbioc_1.20.0
## [13] lazyeval_0.2.0             curl_2.2
## [15] R.utils_2.5.0              R.oo_1.21.0
## [17] preprocessCore_1.36.0      rmarkdown_1.1
## [19] labeling_0.3               splines_3.3.2
## [21] stringr_1.1.0              RCurl_1.95-4.8
## [23] bit_1.1-12                 munsell_0.4.3
## [25] compiler_3.3.2             htmltools_0.3.5
## [27] SummarizedExperiment_1.4.0 gridExtra_2.2.1
## [29] tibble_1.2                 ff_2.2-13
## [31] codetools_0.2-15           XML_3.98-1.5
## [33] bitops_1.0-6               R.methodsS3_1.7.1
## [35] grid_3.3.2                 jsonlite_1.1
## [37] gtable_0.2.0               DBI_0.5-1
## [39] magrittr_1.5               scales_0.4.1
```

```
## [41] stringi_1.1.2           pbapply_1.3-1
## [43] reshape2_1.4.2          viridis_0.3.4
## [45] affyio_1.44.0           xml2_1.0.0
## [47] RColorBrewer_1.1-2      tools_3.3.2
## [49] yaml_2.1.14             AnnotationDbi_1.36.0
## [51] colorspace_1.3-0        GenomicRanges_1.26.1
## [53] affxparser_1.46.0       knitr_1.15
```

8.2 Python and packages

```
python3 -V
```

```
## Python 3.4.5 :: Anaconda custom (64-bit)
```

Only directly used packages are cobra and pandas

```
python3 -c "import cobra; print('cobra', cobra.__version__)"
python3 -c "import pandas; print('pandas', pandas.__version__)"
```

```
## cobra 0.5.4
## pandas 0.19.1
```
